# CXCL12 alone is enough to Reprogram Normal Fibroblasts into Cancer-Associated Fibroblasts

**DOI:** 10.1038/s41420-025-02420-0

**Published:** 2025-04-08

**Authors:** Zelong Ma, Diping Yu, Siqi Tan, Hao Li, Faxiao Zhou, Lei Qiu, Xiaoli Xie, Xiaoming Wu

**Affiliations:** 1 Laboratory of Molecular Genetics of Aging & Tumor, Medical School, Kunming University of Science and Technology, Chenggong Campus, 727 South Jingming Road, Kunming, Yunnan 650500 China; 2 Department of Pathology, Pu’er People’s Hospital, Pu’er, Yunnan 665000 China

**Keywords:** Cancer microenvironment, Non-small-cell lung cancer, Cell invasion, Cell signalling, Phosphorylation

## Abstract

Cancer-associated fibroblasts (CAFs) are critical components of the tumor microenvironment (TME), playing significant roles in regulating cancer progression. However, the underlying mechanism of CAFs activation remains elusive. In this study, we aim to investigates the mechanisms by which CAFs promote the conversion of normal fibroblasts (NFs) to CAFs in lung cancer, with a focus on the role of p53 mutations and the CXCL12/STAT3 signaling axis. We found that CAFs significantly induced NFs to acquire CAFs properties (called CEFs), including upregulation of α-SMA and Vimentin, enhanced proliferation and migration, and increased ability to promote lung cancer cell migration. In vivo, CEFs accelerated A549 xenograft growth and induced spontaneous lung metastasis. CXCL12 was identified as a key factor in NFs-to-CEFs conversion, with its expression positively correlated with CAFs markers in lung cancer. Further investigation confirmed that CXCL12 is sufficient to reprogram NFs into CAFs through the STAT3 pathway. Notably, inhibiting CXCL12 signaling and the STAT3 pathway reduced the conversion of NFs to CAFs, thereby hindering lung cancer progression progression both in vitro and in vivo. Our study reveals CAFs could promote the conversion of NFs to CAFs-like cells through the CXCL12/STAT3 axis, enhancing tumor growth and metastasis in lung cancer. Therefore, inhibition of the CXCL12/STAT3 axis is a promising strategy for the treatment of lung cancers and other CXCL12‐dependent malignancies.

## Introduction

Cancer-associated fibroblasts (CAFs) constitute a major component of the tumor microenvironment (TME), contributing to tumor progression by promoting proliferation, migration, invasion, epithelial-mesenchymal transition (EMT), and drug resistance through the secretion of growth factors, cytokines, and extracellular vesicles, among others [1–3]. CAFs are abundant fibroblasts that acquire α-smooth muscle actin (α-SMA)-positive, activated myofibroblast phenotype [4]. Unlike normal fibroblasts (NFs), CAFs exhibit enhanced proliferative, migratory, and contractile capabilities [5–8]. It is widely acknowledged that CAFs primarily originate from tissue-resident NFs, although the mechanisms underlying the conversion of NFs to CAFs remain controversial.

Accumulating evidence have explored the activation of CAFs through signals derived from both tumor cells and the stromal environment, each contributing unique insights into the complex interplay within the tumor ecosystem. It has been proposed that NFs differentiate into CAFs through education by cancer cells [9]. Previous studies have shown that tumor cells induce fibroblasts from homeostasis to activated states through paracrine signaling molecules, such as transforming growth factor β (TGF-β), platelet-derived growth factor (PDGF), fibroblast growth factor (FGF), and interleukin-1 [10]. Moreover, direct physical contact between cancer cells and fibroblasts through ligand-receptor binding also contributes to CAFs activation [11]. On the other hand, the stroma-derived signals that educate NFs to adopt a pro-tumor CAFs phenotype have also been reported. Neha Saxena found that ECM stiffness can induce mesenchymal stem cells (MSCs) differentiation into CAFs, driving breast cancer invasion and metastasis in TNBC [12]. Furthermore, CAFs educate NFs to facilitate cancer cell spreading and T-cell suppression in gastric cancer [13]. However, the precise mechanisms driving CAFs activation remain largely elusive. Thus, detailed investigations are required to elucidate the molecular mechanisms underlying of CAFs activation, thereby opening a new anti-stroma therapeutic avenue.

The chemokine CXC motif ligand 12 (CXCL12), also known as stromal cell-derived factor-1 (SDF-1), has been shown to be significantly upregulated in many stromal cells and CAFs [14]. By binding to the CXC chemokine receptor 4 (CXCR4, also known as CD184) or CXCR7/ACKR3 [15], CXCL12 activates downstream signaling pathways, including STAT3, NFκB, and MAPK pathways, which are likely responsible for the effects of CXCL12 on cancer aggressiveness [16–20]. Additionally, studies have demonstrated that CXCL12, secreted by CAFs, is crucial for tumor metastasis and immunosuppression, and is positively correlated with poor prognosis [17, 21–23]. However, the role and mechanisms of CXCL12 in CAFs activation within the TME remains unclear.

This study aimed to investigate the mechanism of CAFs activating resident NFs and remodeling TME. We found that CAFs-derived CXCL12 promoted the acquisition of CAF-like properties in NFs both in vitro and in vivo. Furthermore, we discovered that the NFs-to-CAFs conversion can be reversed by blocking CXCL12 signaling or the STAT3 pathway. Our findings provide novel insights into CXCL12-mediated CAFs activation, identifying it as a newly identified mediator in the rapid expansion of pro-tumor fibroblasts.

## Results

### CAFs promote conversion of NFs to CAFs in lung cancer

**Fig. 1:**
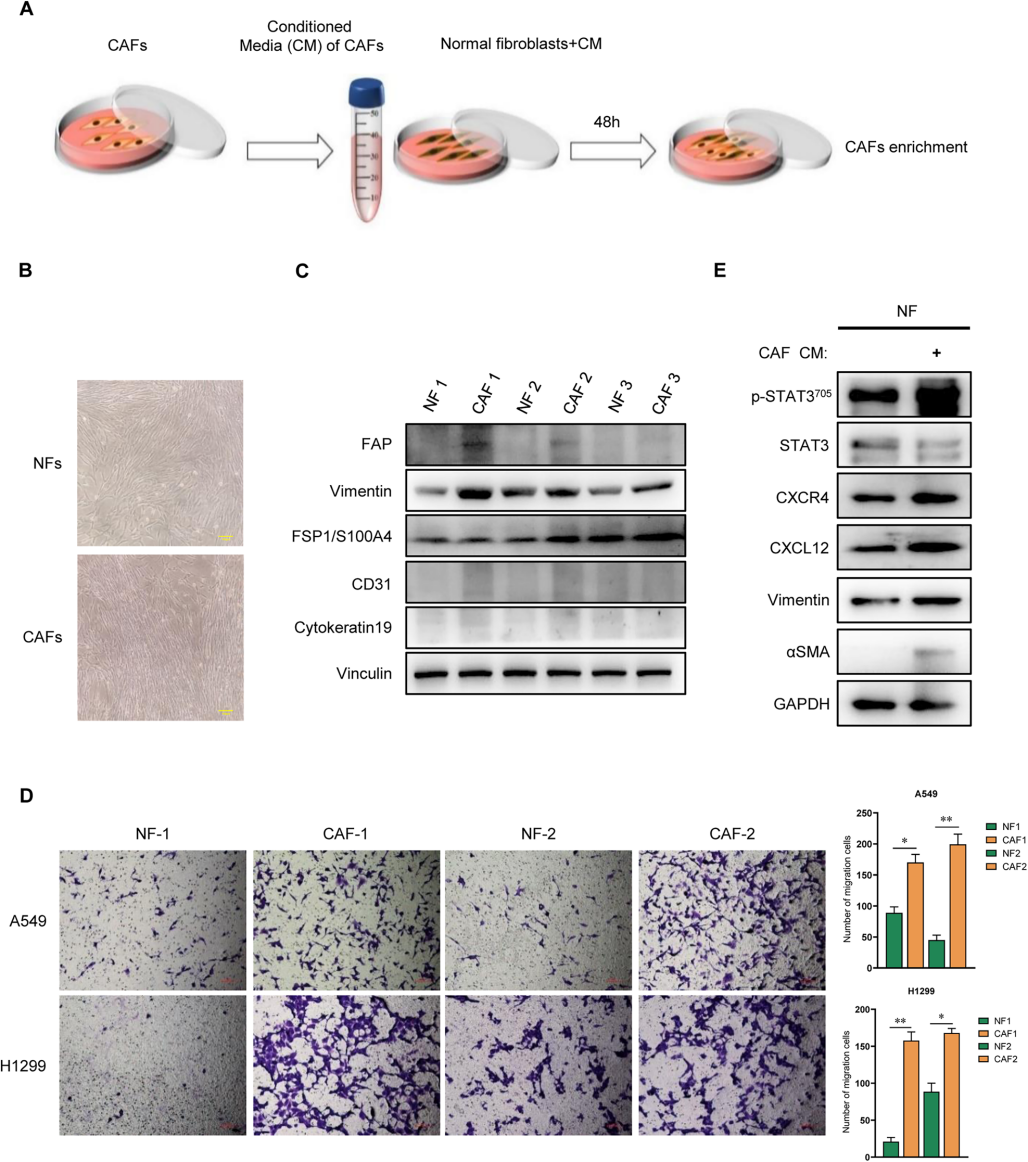
CAFs induce NFs to acquire CAFs properties in lung cancer. **A** Schematic diagram showing the generation of NFs treated with CAFs-CM. **B** Phase-contrast microscopy revealed the typical spindle-like features of fibroblasts in CAFs and NFs isolated from lung cancer tissues, Scale bar = 100 μm. **C** Western blot analysis showed the expression of the fibroblast markers (FAP and Vimentin), the epithelial markers (CK-19) and the endothelial marker (CD31) in CAFs and NFs. **D** The impact of NFs and CAFs on the migratory capabilities of H1299 and A549 was assessed using transwell assay. The results include representative images of cell migration counts (left) and statistical data (right). Data are presented as the mean ± SD of three biological replicates.*, *p* < 0.05; **, p < 0.005. Scale bar = 200 μm. **E** Western blot analysis showed the expression of CXCL12, CXCR4, pSTAT3/STAT3, Vimentin, and α-SMA in NFs treated with DMEM or CAFs CM.

To investigate the clinical impact of CAFs in NFs-CAFs conversion in lung cancer, we isolated pairs of CAFs and NFs respectively from dissected tumor tissues or corresponding para-tumoral non-malignant tissues of several patients with lung cancer. The isolated NFs and CAFs grew well, and the cells showed typical fibroblast morphology (fusiform, fascicular, and scattered) (Fig. 1B). As previously characterized, NFs and CAFs are both positive for fibroblast marker FSP1 while lacking the expression of endothelial marker CD31 and epithelial marker Cytokeratin 19 (Fig. 1C). CAFs specifically exhibit a high expression level of Vimentin and FAP (as known CAFs markers), and exhibited a stronger ability to promote lung cancer cell migration compared to NFs from adjacent tissues (Fig. 1C, D). Then, NFs were treated with CAFs conditioned media (CM), followed by an analysis of CAF-like properties (Fig. 1A). The NFs educated with CAFs CM exhibited significantly higher expression of CAF markers α-SMA and Vimentin (Fig. 1E). These results suggest that CAFs might contribute to confer CAFs-specific properties to NFs.

To explore what causes CAFs activation, we investigated the p53 in CAFs using sequencing. We found that two p53 mutant (p53T55R, p53P72R) was present in CAFs (Figure S1). This result suggests that the alteration of p53 in CAFs might contribute to their specific properties.

### *p53S-CAFs impart CAF-like properties to NFs*

Our previous study demonstrated that p53S activates the CAF-like properties of fibroblasts, thereby promoting cancer cell growth, migration, and invasion [18]. Using normal fibroblasts (NFs, *p53*^*+/+*^), *p53*^*-/-*^ fibroblasts and *p53*^*S/S*^ (p53S) fibroblasts, we further confirmed that p53S fibroblasts display distinct CAF-specific characteristics, which we refer to as “p53S-CAFs” in the paper (Figure S2).

**Fig. 2:**
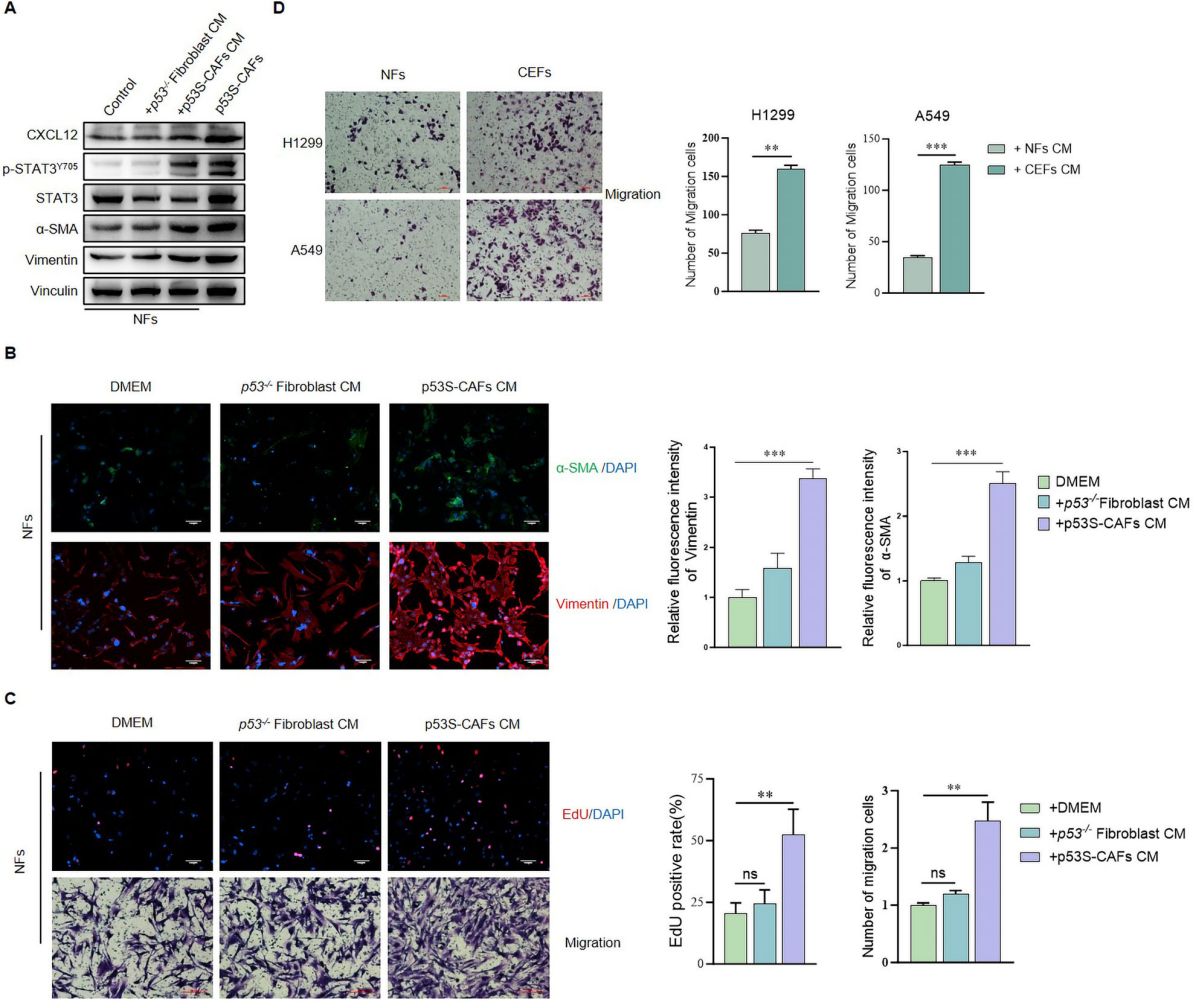
Exposure to p53S-CAFs-CM promotes CAFs activation. **A**. Western blot analysis of the expression of CXCL12, p-STAT3/STAT3, Vimentin, and α-SMA in p53S-CAFs treated with DMEM and NFs treated with DMEM, *p53*^*-/-*^ fibroblast CM, and p53S-CAFs CM. **B** Immunofluorescence analysis of the expression of Vimentin and α-SMA in NFs treated with DMEM, *p53*^*-/-*^ fibroblast CM, and p53S-CAFs CM. **C** Calculation of the percentage of cells that incorporated EdU or the number of migration cells for Transwell migration assay in NFs alone and treated with p53S-CAFs-CM. *, *p* < 0.05. **, *p* < 0.005. **D** Transwell migration assay of lung cancer cells H1299 and A549 cultured with NFs CM or CEFs CM. Average migration ±SEM from three independent experiments performed(*n* = 3). **, *p* < 0.005. ***, *p* < 0.001.

To further test the possibility of CAF-mediated education of NFs, we treated NFs with CM derived from *p53*^*-/-*^ fibroblasts and p53S-CAFs, followed by an analysis of CAF-like properties (Fig. 1A). NFs educated with p53S-CAFs CM exhibited significantly higher expression of CAFs markers, including α-SMA and Vimentin (Fig. 2A, B). Furthermore, p53S-CAFs CM-educated NFs displayed enhanced proliferative and migratory capacities (Fig. 2C), as well as a stronger ability to promote the migration of lung cancer cell lines H1299 and A549 (Fig. 2D). These results showed that p53S-CAFs‐CM‐educated NFs(hereinafter referred to as “CEFs”) displayed CAF-like phenotypes.

**Fig. 3:**
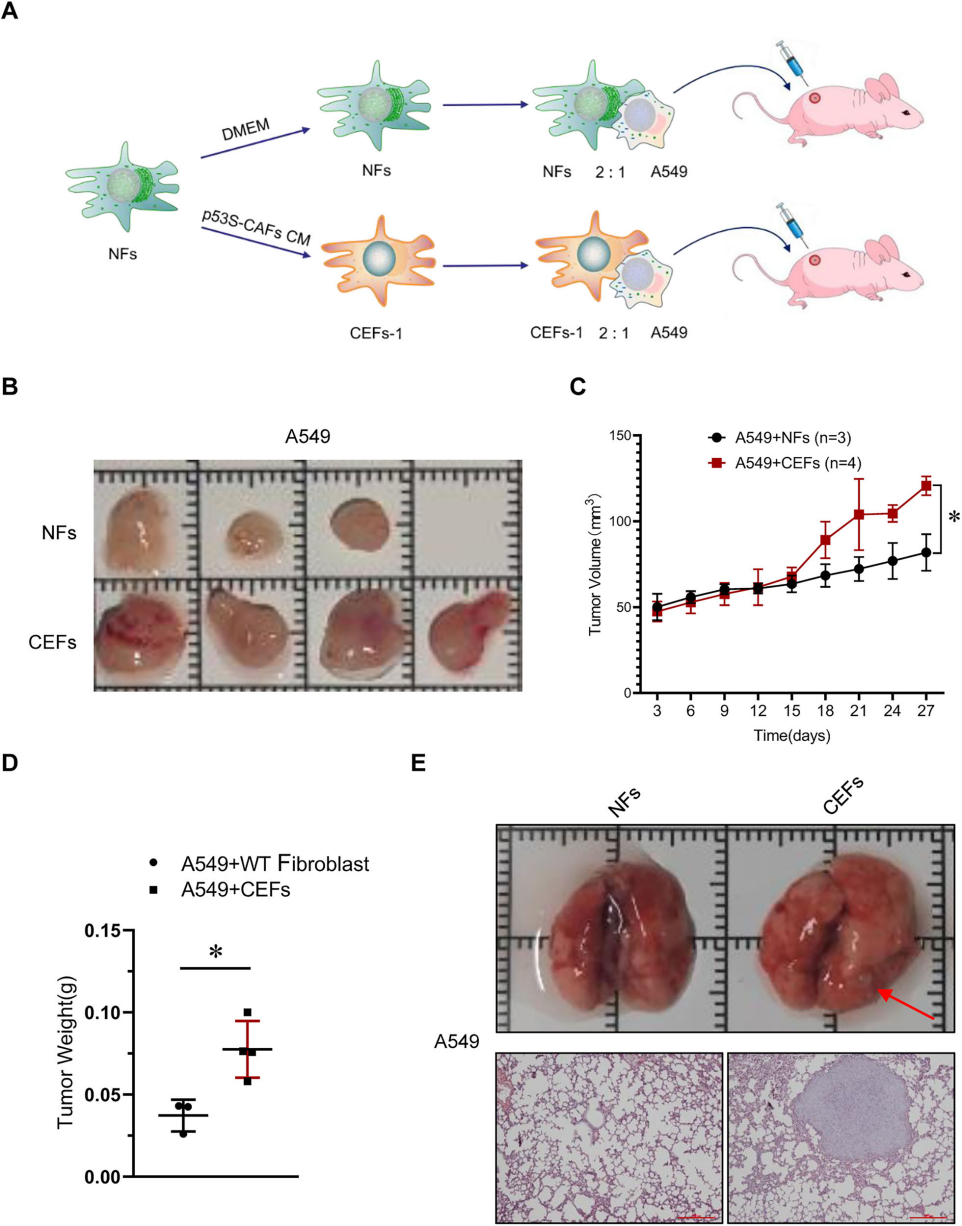
NFs treated with CAFs-CM promoted tumor growth in vivo. **A** The flowchart for the experimental design of xenograft tumor models. **B** Representative images show the xenograft tumors in the backs of nude mice formed by mixed subcutaneous injection of CEFs (or NFs) and A549 cells. **C** Tumor growth graphs indicated the tumor volumes at different time course (*n* = 3 or 4 mice per group). *p < 0.05. **D** Weighing of the tumors was performed upon dissection, *p < 0.05. **E** Representative images show the lung metastasis formed by subcutaneous injection of A549 cells incubated with CEFs (or NFs). Red arrows point to the metastatic nodules. HE staining of representative lung metastatic nodules is shown (scale bar = 100 μm).

To validate these in vitro observations, we assessed the effects of CEFs in a subcutaneous xenograft model using A549 cells. A549 cells mixed with either NFs or CEFs were subcutaneously inoculated into male nude mice, and tumor growth was monitored (Fig. 3A). As shown in Fig. 3B, CEFs significantly accelerated the growth of A549 xenografts. Tumors formed in the presence of CEFs exhibited substantially increased weight and volume compared to those formed with NFs (Fig. 3C, D).

Notably, mice bearing tumors derived from A549 cells mixed with CEFs developed prominent microscopic lung metastases, whereas tumors formed with A549 cells and NFs did not show such metastases (Fig. 3E). The presence of metastatic tumor nodules in the lungs was further confirmed by H&E staining (Fig. 3E). These results suggest that CEFs are highly potent in promoting A549 xenograft growth and inducing spontaneous metastatic dissemination.

### CAFs‐derived CXCL12 is sufficient to reprogram NFs to CEFs

**Fig. 4:**
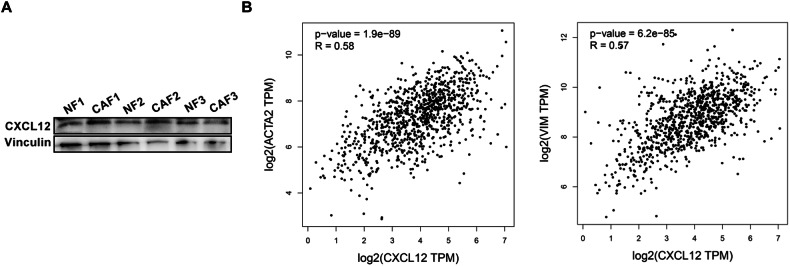
CXCL12 expression positively correlated with CAF markers in lung cancer. **A** Western blot analysis of the expression of CXCL12 in NFs and CAFs. **B** Correlation of CXCL12 expression with CAFs markers α-SMA and Vimentin (*P* < 0.001, *R* > 0).

The role of CAF-derived CM in the conversion of NFs to CEFs was further investigated. Western blotting confirmed increased CXCL12 protein expression in p53S CAFs (Fig. 2B). CXCL12 was also significantly increased in CAF1 and CAF2 compared with NF1 and NF2, respectively (Fig. 4A). Higher CXCL12 expression was associated with poor prognosis in many types of tumors, inluding lung cancer. Moreover, there was a significantly positive correlation between CXCL12 expression and CAFs markers α-SMA and Vimentin in lung cancer (Fig. 4B, C). It is likely that the high CXCL12 expression levels promote conversion of NFs to CAFs.

**Fig. 5:**
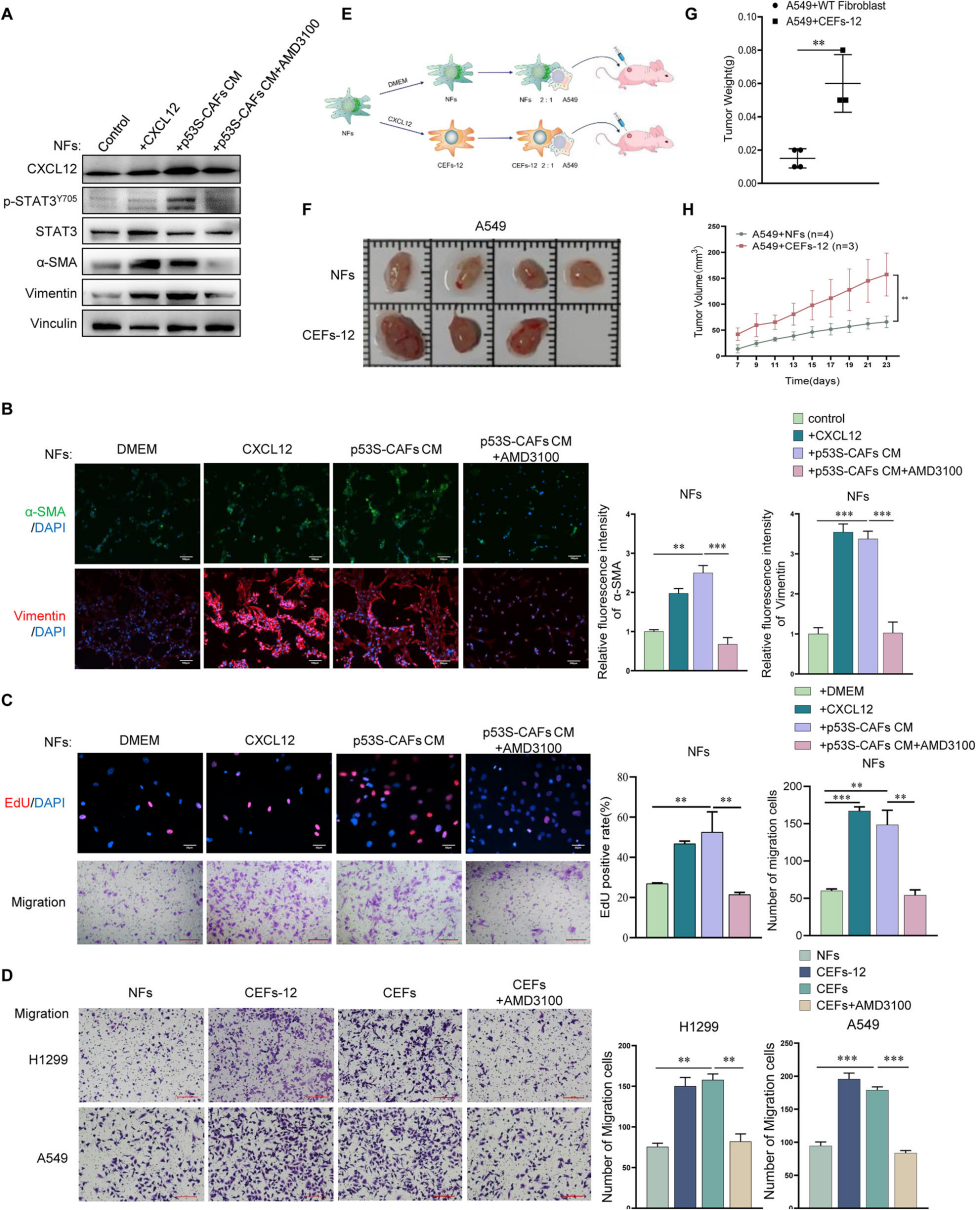
CAFs-derived CXCL12 promote NFs conversion into CAFs. **A** Western blotting analysis was conducted on NFs that were either untreated or treated with CXCL12, p53S-CAFs-CM, or a combination of p53S-CAFs-CM and AMD3100. **B** EdU incorporation assay of NFs treated with DMEM, CXCL12, CXCL12 and AMD3100, p53S-CAFs CM, p53S-CAFs CM and AMD3100. Representative images (left) and statistics (right) of the percentage of cells that incorporated EdU were shown. **, *p* < 0.005. **C** Transwell assay of NFs treated with DMEM, CXCL12, CXCL12 and AMD3100, p53S-CAFs CM, p53S-CAFs CM and AMD3100. Representative images (left) and statistics (right) of migration assay were shown. **, *p* < 0.005. ***, *p* < 0.001. **D** Transwell co-culture assay was employed to evaluate the pro-migratory capacity of normal fibroblasts, CEFs-12 (normal fibroblasts treated with CXCL12), CEFs (normal fibroblasts treated with p53S-CAFs conditioned medium), and CEFs+AMD3100 towards lung cancer cells H1299 and A549. The results include representative images of cell migration (left) and statistical data (right). **, *p* < 0.005, ***, *p* < 0.0005. Scale bar = 200 μm. **E** The flowchart for the experimental design of xenograft tumor models. **F** Representative images show the xenograft tumors in the backs of nude mice formed by mixed subcutaneous injection of CEFs-12 (or NFs) and A549 cells. **G** Tumor growth graphs indicated the tumor volumes at different time course (*n* = 3 or 4 mice per group). **p < 0.01. **H** Precise weighing of the tumors was performed upon dissection, **p < 0.01.

To detect whether the potential role of CXCL12 in mediating the conversion of NFs to CAFs, exogenous CXCL12 was added directly to the CM of NFs, and CAF-like characteristics were analyzed. As shown in Fig. 5A, B, CXCL12 treatment significantly upregulated the expression of α-SMA and Vimentin in NFs. Additionally, CXCL12 promoted NFs proliferation and migration, mirroring the effects of p53S-CAFs-derived CM (Fig. 5C). NFs treated with CXCL12 are hereafter referred to as “CEFs-12.”

Further experiments revealed that CEFs-12 significantly enhanced the migratory abilities of lung cancer cell lines H1299 and A549 (Fig. 5D). Consistent with the observations in lung cancer cells H1299 and A549, CXCL12 signaling is involved in the NFs-to-CAFs transition, which promotes cancer cells Hela and U2OS metastasis (figure S3).

To evaluate the role of CXCL12 in NFs-to-CEFs conversion in vivo, A549 cells were subcutaneously co-injected with either NFs or CEFs-12 into male nude mice (Fig. 5E). Results demonstrated that co-injection with CEFs-12 markedly accelerated A549 tumor growth compared to co-injection with NFs (Fig. 5F–H). Importantly, the CXCL12 antagonist AMD3100 effectively inhibited these effects (Figs. 5C, 7A).

**Fig. 6:**
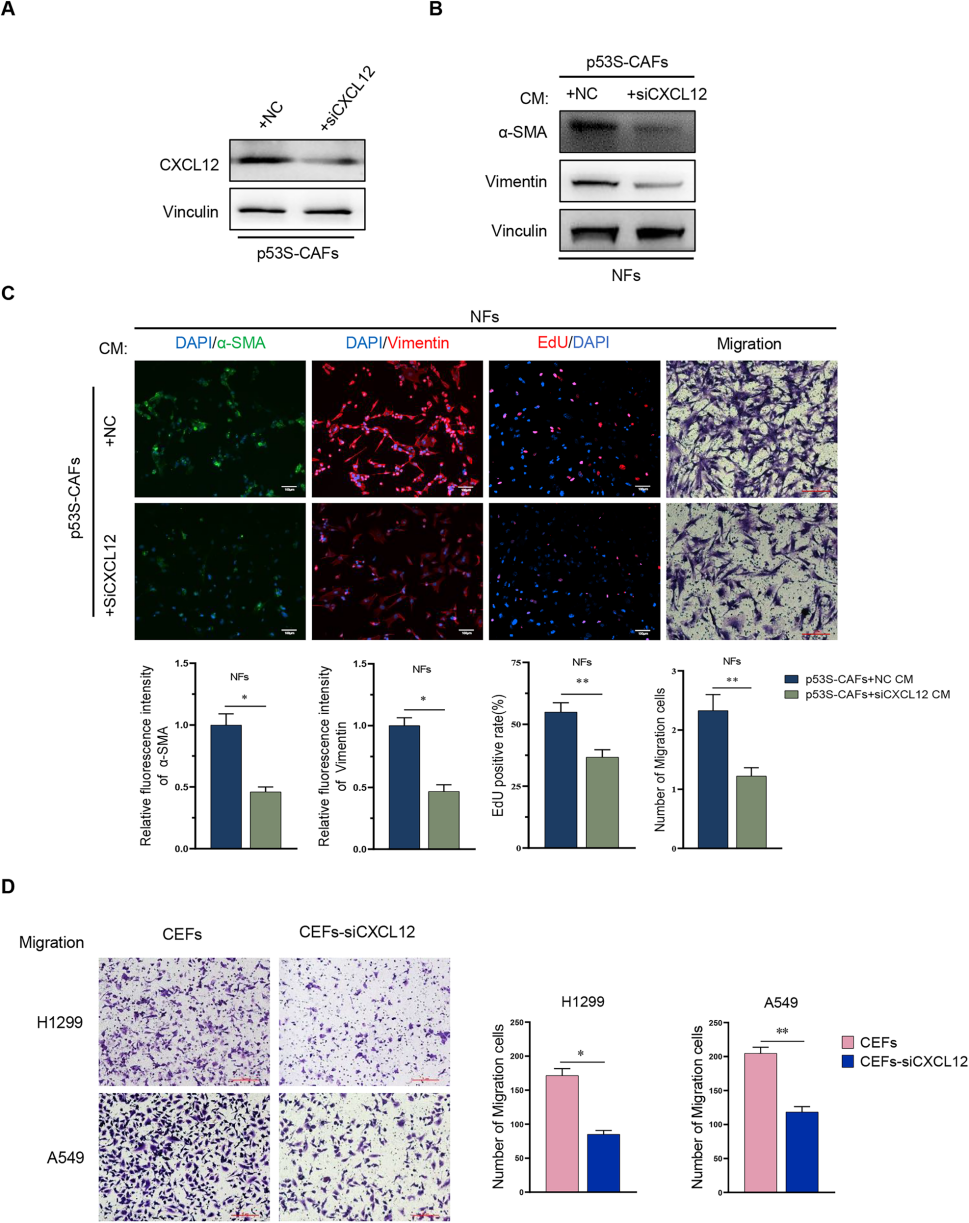
Knockdown of CXCL12 reverses the role of CAFs in promoting NFs-CAFs conversion. **A** Western blot analysis of the knockdown efficiency following the transfection of CXCL12 siRNA into p53S-CAFs. **B** Western blot assessment of the impact on the expression of CAFs marker proteins in normal fibroblasts by conditioned medium after CXCL12 knockdown in p53S-CAFs. **C** Immunofluorescence analysis was conducted on the expression of CAFs markers α-SMA and Vimentin in *p53*^*+/+*^ fibroblasts treated with either p53S-CAFs+NC CM or p53S-CAFs+siCXCL12 CM, in addition to the percentage of EdU-incorporated positive population. *p < 0.05, **p < 0.005. **D** The impact of CEFs and CEFs+siCXCL12 on the migratory capabilities of H1299 and A549 was assessed using transwell assay. The results include representative images of cell migration counts (left) and statistical data (right). *p < 0.05; **p < 0.005. Scale bar = 200 μm.

To further confirm the involvement of CXCL12 in p53S-CAFs-mediated activation of NFs, p53S-CAFs were transfected with CXCL12-specific siRNA. The knockdown efficiency was confirmed by assessing CXCL12 expression levels in p53S-CAFs (Fig. 6A). CXCL12 knockdown significantly reduced the p53S-CAF-derived CM-induced expression of α-SMA and Vimentin in NFs (Fig. 6B). Moreover, CXCL12 knockdown reversed the enhanced proliferation and migration of NFs induced by p53S-CAFs-derived CM (Fig. 6C).

Critically, CEFs generated using CM from CXCL12-knockdown p53S-CAFs (referred to as CEFs-SiCXCL12) showed significantly reduced ability to promote H1299 and A549 migration compared to CEFs derived from p53S-CAFs-NC CM (NFs pretreated with p53S-CAFs transfected with a negative control siRNA) (Fig. 6D). These findings indicate that CXCL12 in the CM of p53S-CAFs is a key mediator of the conversion of NFs to CEFs and their subsequent pro-tumorigenic effects.

### CXCL12 induces NFs-CEFs conversion by upregulating STAT3 pathway

**Fig. 7:**
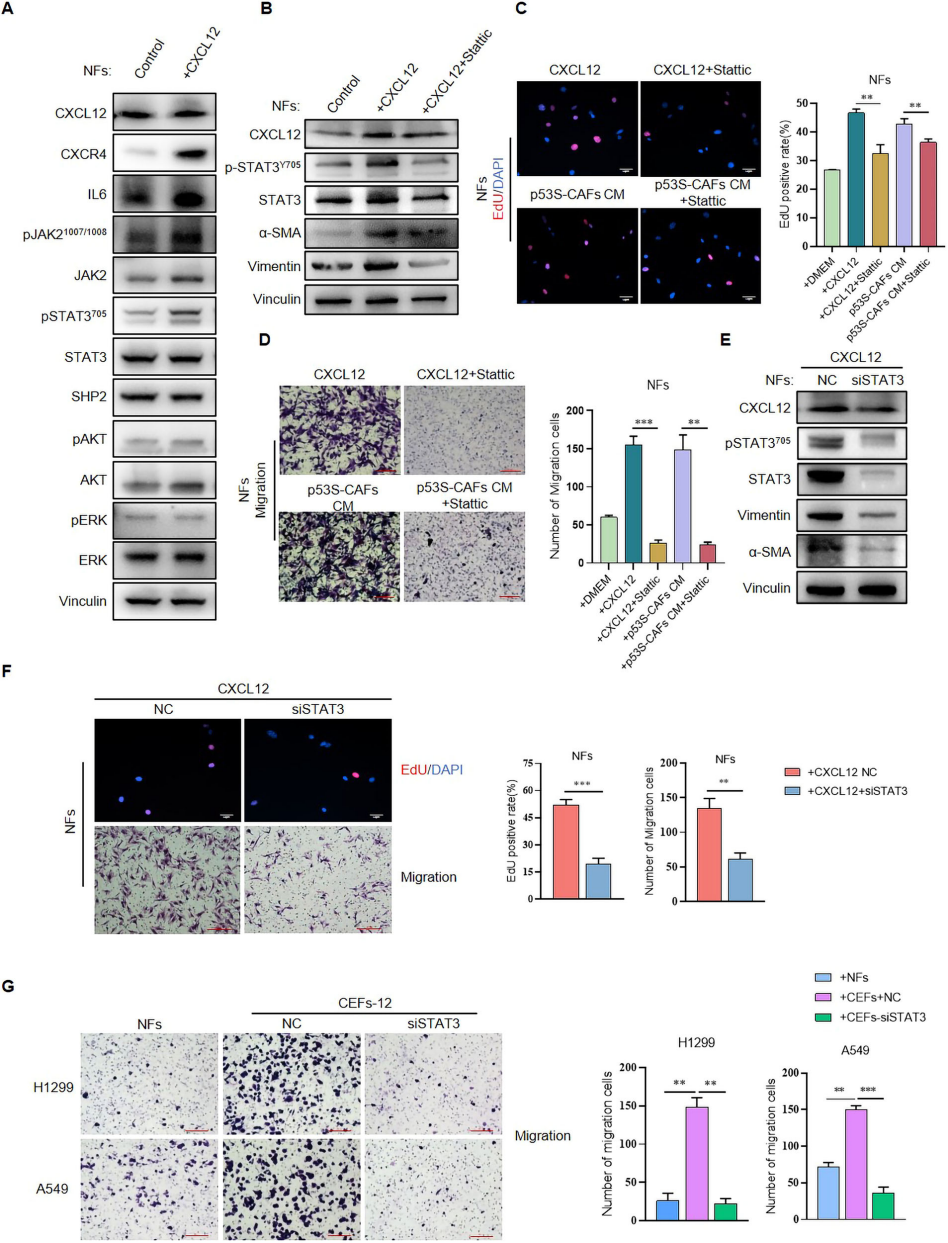
p53S-CAFs-derived CXCL12 activated STAT3 pathway through the CXCR4. **A** p53S-CAFs-derived CXCL12 activated STAT3 pathway. NFs were treated with CXCL12 (20 ng/ml, 24 hours). The protein levels of CXCL12, CXCR4, IL6, p-JAK2, JAK2, p-STAT3, STAT3, SHP2, α-SMA, vimentin, p-ERK, ERK, p-AKT and AKT were detected by Western blot. **B** Western blot analysis of CXCL12, p-STAT3, STAT3, α-SMA and Vimentin from NFs alone and treated with CXCL12, or CXCL12 and Stattic. **C** Proliferative ability of NFs treated with CXCL12, CXCL12 and Stattic (upper), or p53S-CAFs CM, p53S-CAFs CM and Stattic(down) were measured by EdU incorporation assay. **, *p* < 0.005. **D** Migratory ability of NFs treated with CXCL12, CXCL12 and Stattic (upper), or p53S-CAFs CM, p53S-CAFs CM and Stattic(down) were measured by transwell migration assay. **, *p* < 0.005. ***, *p* < 0.001. Scale bar = 200 μm. **E** NFs were treated with CXCL12 and then transfected with STAT3 siRNA or control siRNA. STAT3 and p-STAT3 expression was measured by Western blot. *, *p* < 0.05. **, *p* < 0.005. **F** Proliferative and Migratory ability were measured by EdU incorporation assay and transwell assay. **, *p* < 0.005. ***, *p* < 0.001. **G** CEFs (NFs were treated with CXCL12) were transfected with STAT3 siRNA or control siRNA. Transwell assays detected the migration ability of H1299 and A549 co-cultured with CEFs or CEFs-SiSTAT3. **, *p* < 0.005. ***, *p* < 0.001. Scale bar = 200 μm.

CXCL12 increased CXCR4 expression in NFs (Fig. 7A). The CXCL12/CXCR4 axis activates various signaling pathways in the tumor microenvironment, including the PI3K/AKT, MAPK/ERK, and JAK2/STAT3 pathways [24]. Therefore, we further investigated which pathway CXCL12 utilizes to mediate the conversion of NFs into CEFs.

CXCL12 treatment did not affect the phosphorylation of AKT or ERK in NFs (Fig. 7A). However, CXCL12 and p53S-CAFs-CM increased the phosphorylation of STAT3 (Tyr705) (Figs. 5A, 7A), an effect that could be attenuated by the addition of AMD3100. Phosphorylation of STAT3 (Tyr705) indicates activation of the STAT3 pathway, which has been reported to significantly contribute to CAFs activation [25–28]. Furthermore, the expression of the positive regulator of p-STAT3, IL6 and p-JAK2 (Tyr1007/1008), was significantly upregulated, whereas the expression of the negative regulator SHP2 was not affected (Fig. 7A). These results suggest that the CXCL12/STAT3 axis may be involved in the pro-CAFs activation effect of p53S-CAFs.

To further confirm whether CXCL12 induces NFs-to-CEFs conversion through the STAT3 pathway, we treated NFs with CXCL12 in the presence or absence of Stattic, a specific inhibitor of p-STAT3. The inhibitory effects were detected by Western blotting (Fig. 6B). Compared to NFs treated with CXCL12 alone, those exposed to both CXCL12 and Stattic exhibited downregulated expression of CAFs markers α-SMA and Vimentin (Fig. 6B). Additionally, altered proliferation and migration abilities were observed in these groups (Fig. 7C, D).

To rule out off-target effects of Stattic, we knocked down STAT3 in NFs using siRNA. Cells exposed to CXCL12 for 24 hours were subsequently transfected with siSTAT3 prior to conducting Western blotting, proliferation, and transwell migration assays. STAT3 knockdown repressed the expression of α-SMA and Vimentin induced by CXCL12 (Fig. 7E). Furthermore, STAT3 knockdown inhibited the proliferation and migration of NFs induced by CXCL12 (Fig. 7F). Importantly, the promotive effects of CXCL12-educated NFs (CEFs-12) on the migration of lung cancer cells (H1299 and A549) were decreased in CEFs with STAT3 knockdown (Fig. 7G).

**Fig. 8:**
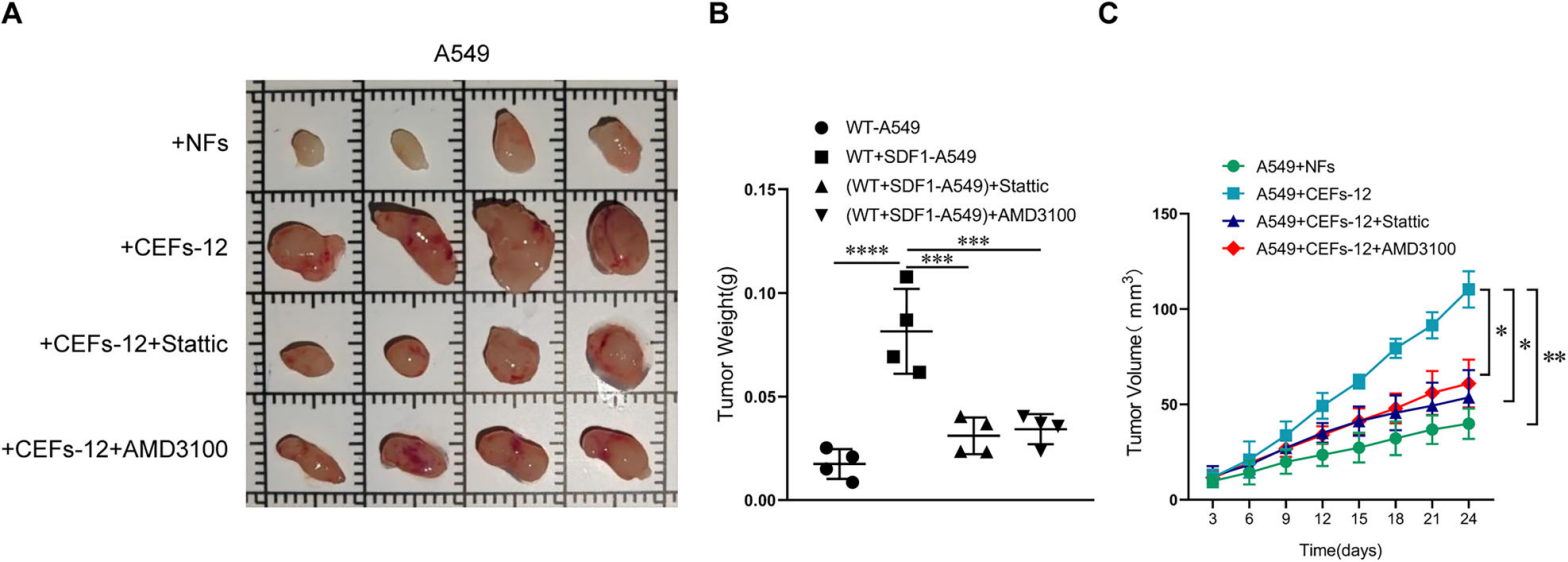
Stattic and AMD3100 attenuated the ability of CXCL12-activated NFs to promote tumor growth in vivo. **A** Representative images show the xenograft tumors in the backs of nude mice formed by mixed subcutaneous injection of CEFs-12 (or NFs) and A549 cells, with regular intraperitoneal injections of saline, AMD3100, or Stattic. *n* = 4 mice per group. **B** Precise weighing of the tumors was performed upon dissection, ***, *p* < 0.001. **C** Tumor growth graphs indicated the tumor volumes at different time course (*n* = 4 mice per group). *, *p* < 0.05, **, *p* < 0.01.

Finally, we sought to extend these results to an in vivo model system by subcutaneously implanting nude mice with A549 cells mixed with either NFs or CEFs-12. Tumors mixed with CEFs-12 exhibited significantly enhanced growth relative to those mixed with NFs, and both AMD3100 and Stattic significantly reduced tumor growth (Fig. 8A–C).

In summary, these findings reveal CAFs could contribute to reprogramming of NFs into CAFs via CXCL12. CXCL12 acts through the STAT3 pathway to activate CAFs characteristics in NFs. Therefore, inhibition of this axis is a promising strategy for the treatment of Lung cancers and other CXCL12‐dependent malignancies.

## Discussion

Numerous studies have provided compelling evidence that CAFs play a critical role in cancer development and the response to cancer treatment [19, 20]. However, the mechanisms by which NFs are transformed into CAFs remain poorly understood. Previous reports have indicated that CAFs activation is mediated by cancer cells [29]. Interestingly, our study uncovers a novel mechanism for the generation of CAFs in the absence of cancer cells. We demonstrated that CXCL12 secreted by p53S-CAFs acts locally on adjacent NFs, activating the STAT3 signaling pathway. This ultimately induces a CAF-like phenotype in NFs, which we term CAF-like fibroblasts (CEFs). In conclusion, our findings describe a CXCL12/STAT3 signaling axis that mediates the activation of CAFs by stromal p53S mutants and propose a new therapeutic strategy targeting CXCL12/STAT3 in CAFs to improve cancer treatment outcomes.

We identified two p53 mutations (p53T55R and p53P72R) in CAFs (Figure.  S1). However, how these mutations influence CAFs activation and their role in lung cancer progression remains to be explored. Moreover, with the increase in passage numbers, the isolated NFs gradually exhibited elevated expression levels of α-SMA and Vimentin (not shown in the paper, [29]), which poses a challenge that we must address.

Increasing evidence suggests that CAFs secrete CXCL12 and promote cancer progression through the CXCR4 receptor [30]. Activation of the CXCL12-CXCR4 axis is prevalent in many solid tumors and contributes to tumor immunosuppression and metastasis [25]. However, the role of CXCL12 in the activation of CAFs remains unexplored. We demonstrated that CXCL12 is critical for p53S-CAFs-mediated NFs-to-CAFs conversion. Specifically, we observed high levels of CXCL12 expression in p53S-CAFs, which upregulate CXCR4 expression in NFs. Similar to the CM of p53S-CAFs, CXCL12 induced NFs activation, resulting in increased expression of CAFs markers (e.g., α-SMA and vimentin), as well as enhanced cell proliferation and migration. Notably, CEFs/CEFs-12, an activated form of NFs induced by p53S-CAFs or CXCL12, lost their CAF-like phenotype upon CXCL12 knockdown or treatment with the CXCL12 inhibitor AMD3100. These findings indicate that CXCL12 secreted by p53S-CAFs is closely associated with the CAF-like phenotype in NFs.

Previous studies have shown that the STAT3 pathway plays tumor-promoting roles in various malignancies [31]. It has been reported that the STAT3 pathway enhances the activation and proliferation of fibroblasts [24, 26]. For instance, IL-1 activates the STAT3 pathway to shape CAFs heterogeneity in pancreatic ductal adenocarcinoma [27]. Consistently, our results revealed that STAT3 signaling is significantly activated in CXCL12-mediated NFs activation. We demonstrated that CXCL12 activates normal fibroblasts in a STAT3-dependent manner.

In addition to the phenotypic changes in CAFs, a well-recognized functional characteristic of CAFs is their supportive role in tumor progression. In our study, CEFs significantly enhanced lung cancer cell migration and growth both in vitro and in vivo. Blocking the STAT3 pathway in CEFs markedly reduced their tumor-promoting effects on lung cancer cells.

Despite these findings, our study has certain limitations. The precise mechanism by which CXCL12 regulates the STAT3 pathway requires further elucidation. Recent research has demonstrated that CXCL12 signaling can trigger the STAT3 pathway. For example, in the pre-metastatic microenvironment of hepatocellular carcinoma, CXCL12 binds to CXCR4 and, under the mediation of Prrx1, promotes nuclear translocation and phosphorylation of STAT3, thereby activating STAT3 signaling [28].

The STAT3 pathway is essential for the pro-tumorigenic functions of CAFs [18, 32]. These observations underscore the need for further investigation into the effects of CXCL12 on tumor stromal cells. In studies of tumor cells, CXCL12 binding to its receptor can activate various downstream signaling pathways, including the STAT3 signaling pathway, the MAPK signaling pathway, the PI3K/AKT/NF-κB signaling axis, the MEK/ERK pathway, and the Wnt/β-catenin pathway. Among these, studies have shown that STAT3 can regulate the expression of NF-κB and may have feedback mechanisms with ERK, β-catenin, and AKT. This provides insights into the mechanisms of the CXCL12-STAT3 axis in fibroblast research [33].

In recent years, targeting CAFs has emerged as a novel therapeutic strategy in cancer treatment [33]. Innovative approaches combining CAF-targeted and cancer cell-targeted therapies may hold significant promise. Targeting the CXCL12/STAT3 axis could have dual benefits: decreasing the population of α-SMA-positive CAFs and reducing the secretion of tumor-promoting cytokines and chemokines.

In summary, our study elucidates a novel molecular mechanism underlying the crosstalk between NFs and CAFs in promoting cancer cells metastasis. The identification of the CXCL12/ STAT3 pathway as a key participant in the NFs-to-CAFs transition highlights critical therapeutic targets for developing novel cancer treatments.

## Methods and material

### Cell lines and Treatment

As described previously [34], we harvested MEFs with different genotypes in E13.5 days and cultured in DMEM (Gibco) with 10%(v/v) heat-inactivated fetal bovine serum(FBS) (Hyclone, Logan, UT, USA). Briefly, embryos were rinsed several times with sterile phosphate-buffered saline (PBS), minced with a scalpel and digested with trypsin (0.25%) for 10 min at 37 °C, following removal of the head and liver. The trypsin was inactivated by addition of DMEM supplemented with 10% FBS, the embryo was plated into one 10 cm dish. The genotypes of MEFs were *p53*^*+/+*^ (normal fibroblast), *p53*^*-/-*^, *p53*^*S/S*^. All cell lines were authenticated at the beginning of the planned experiments by genotype analysis. *p53*^*S/S*^ MEFs were referred as p53S-CAFs in the paper. Murine lung cancer cell (LLC), human H1299 and A549 cell, Hela and U2OS were purchased from China Infrastructure of Cell Line Resources and cultured in RPMI-1640 supplemented with 10% FBS. All cells were cultured at 37 °C with 5% CO_2_ and 3% O_2_. Routine tests for cell mycoplasma infection were perform and confirmed to be negative.

Recombinant human CXCL12 protein was purchased from Sino Biological (Beijing, China). CXCL12 inhibitor AMD3100 and STAT3 pathway inhibitor Stattic were purchased from MCE company (NJ, USA).

### Isolation and Culture of Patient-Derived Cancer-Associated Fibroblasts (CAFs)

Human CAFs and NFs were isolated from lung adenocarcinoma tissues and adjacent normal lung tissues, which were obtained from three patients. These patients underwent surgical resection at Pu’er People’s Hospital (Yunnan, China) in 2023. Tumor tissues were cut into 1–2 mm blocks, digested with trypsin and 0.5% collagenase at 37 °C for 1 hour, and seeded in T25 culture flasks containing 5 mL DMEM with 10% FBS. After 72 hours, the medium was changed to remove non-adherent cells. The CAFs and NFs were passaged for up to 5‐7 populations in the subsequent experiments. All cell cultures were maintained at 37 °C with 5% CO_2_ and 3% O_2_, and mycoplasma contamination was routinely tested and confirmed negative.

### Transient siRNA transfection

Fibroblasts were treated with CXCL12(48 h) and transfected with siRNA (siSTAT3 sequences, SiSTAT3-1: 5’-CUGGAUAACUUCAUUAGCA-3’). Random sequences were used as a negative control (NC) using EL transfection reagent (TransGen Biotech, Beijing, China). Then, 48 h later, cells were collected for Western blot analyses to determine knockdown efficiency. In addition, the siRNA sequences specific for CXCL12 were:

siCXCL12–1, 5′-CUCGGUUGCAGUUCGUAGA-3′.

### Conditioned medium (CM) collection

The *p53*^*-/-*^ fibroblast and p53S CAFs were seeded into 10-cm plates at identical density. The culture medium was collected 48 h, remove suspended cells with 0.45μm filter, and stored in −80 °C until Use.

p53S CAFs-CM or CXCL12 treated normal fibroblasts (48 h) were referred as CEFs or CEFs-12 in this study.

### Western blotting

Fibroblasts were lysed in a RIPA buffer containing Protease Inhibitor Cocktail (Roche). Sample proteins (15 μg) were separated by SDS-PAGE and then transferred to PVDF membranes. After blocking in 5% non-fat milk or 2% bovine serum albumin (BSA) for 2 h at room temperature, membranes were incubated with primary antibodies overnight at 4 °C. The membranes were then incubated with horseradish peroxidase-labeled secondary antibodies and visualized with ECL. The following primary antibodies were used: anti-p-STAT3(Tyr705) (1:1000, 9145S, CST, USA), anti-STAT3 (1:1000, 4904S, CST, USA), anti-p53 (1:1000, ab26, Abcam, UK), anti-α-SMA (1:1000, A17910, Abclonal, China), anti-Vimentin (1:1000, A19607, Abclonal, China), anti-p-JAK21007/1008 (1:1000, 3771S, CST, USA), anti-JAK2 (1:1000, 4040S, CST, USA), anti-SH-PTP2 (1:1000, sc-7384, Santa, USA), anti-SDF1 (1:1000, sc-74271, Santa, USA), anti-CXCR4 (1:1000, ab124824, Abcam, UK), anti-IL6 (1:1000, A11115, Abclonal, China), anti-p-AKT (Ser473) (1:1000, 4060S, CST, USA), anti- AKT (1:1000, 9272S, CST, USA), anti-p-ERK1/2 (1:1000, AP0235, Abclonal, China), anti-ERK1/2 (1:1000, WL01864, Wanleibio, China), and anti-Vinculin (1:2000, bsm-54148R, Bioss, China). The relative expression was normalized to Vinculin expression.

### EdU incorporation assays

EdU assay was conducted using the Apollo567 in vitro imaging kit (RiboBio Corporation, Guangzhou, China) following the manufacturer’s protocol. After 4 h treatment with EdU (5 μmol/L), fibroblasts were fixed with 4% paraformaldehyde, permeabilized with 0.3% Triton X-100, and co-stained with Apollo fluorescent dyes and DAPI (5 μg/ml). The EdU-positive cells were recorded and calculated under fluorescence microscopy.

### Transwell assays

Transwell assays were used to evaluate the migration capacity of fibroblasts. Cells suspension was seeded to the upper chamber of non-coated Transwell plates for migration assays; DMEM, p53S-CAFs-CM supplemented with 10% FBS or CEFs was added to the lower chambers. The migrated cells were fixed, stained, and photographed under light microscopy.

### Xenograft Experiments

Male BALB/c nude mice (6–8 weeks) were purchased from Yunnan University Laboratory Animal Center (Kunming, China). The BALB/c nude mice were randomly divided into groups of 3 or 4 mice each for the experiment. A549 cells (1×10^6^) mixed with NFs or CEFs (1:2) were injected subcutaneously into the left or right side of BALB/c nude mice. Every other day, intraperitoneal injections of either physiological saline, AMD3100, or Stattic were administered, the mice were regularly monitored for growth and subcutaneous tumor volume was measured. Tumor volumes were calculated using the formula volume (mm^3^) = L × W^2^ / 2 (length L, mm; width W, mm).

### H&E staining

Tumor tissue from mice was fixed with formalin, dehydrated with graded ethanol, and embedded in paraffin after transparentizing with xylene. Tissue sections were cut to 4 μm and then deparaffinized with xylene and rehydrated with graded ethanol. Sections were stained with haematoxylin for 10 minutes, differentiated with 1% ethanol hydrochloric acid for 10 seconds, treated with 1% ammonia water for 15 seconds, and then stained with eosin for 2 minutes. The tissue sections were then dehydrated with graded ethanol, transparentized with xylene, sealed with neutral resin, and observed under a microscope. Images were obtained and analysed under a microscope (Nikon, Japan).

### Statistical analysis

Experiments were performed in triplicate and repeated at least three times. Statistical analysis was performed using GraphPad Prism (GraphPad Software, San Diego, CA, USA), and all data are presented as the mean ± standard deviation (SD). The difference between the two groups was analyzed using an unpaired Student’s t test for the data was normally distributed. The difference among multiple groups was analyzed by one-way analysis of variance (ANOVA) for the data with homogeneity of variance. *P* < 0.05 was considered significant.

GEPIA2 (http://gepia2.cancer-pku.cn/) enables comparison of the differentially expressed genes based on tumor and normal samples from the TCGA databases [35]. By using the “Correlation Analysis” module in GEPIA2, we analyzed the correlation between CXCL12 expression and CAFs markers. Spearman correlation analysis was used to assess the significance of correlations.

## Date availability

The datasets used and/or analyzed during the current study are available from the corresponding author on reasonable request.

## Supplementary information

Supplementary Figure

Original Images for BlotsGels

## References

[1] Chen X, Song E. Turning foes to friends: targeting cancer-associated fibroblasts. Nat Rev Drug Discov. 2019;18:99–115. 30470818 10.1038/s41573-018-0004-1

[2] Biffi G, Tuveson DA. Diversity and Biology of Cancer-Associated Fibroblasts. Physiol Rev. 2021;101:147–76. 32466724 10.1152/physrev.00048.2019 PMC7864232

[3] Kobayashi H, Enomoto A, Woods SL, Burt AD, Takahashi M, Worthley DL. Cancer-associated fibroblasts in gastrointestinal cancer. Nat Rev Gastroenterol Hepatol. 2019;16:282–95. 30778141 10.1038/s41575-019-0115-0

[4] Hanley CJ, Mellone M, Ford K, Thirdborough SM, Mellows T, Frampton SJ, et al. Targeting the Myofibroblastic Cancer-Associated Fibroblast Phenotype Through Inhibition of NOX4. Jnci-J Natl Cancer Inst. 2018;110:109–20. 10.1093/jnci/djx121 PMC5903651 28922779

[5] Kalluri R. The biology and function of fibroblasts in cancer. Nat Rev Cancer. 2016;16:582–98. 27550820 10.1038/nrc.2016.73

[6] Mishra PJ, Mishra PJ, Humeniuk R, Medina DJ, Alexe G, Mesirov JP, et al. Carcinoma-associated fibroblast-like differentiation of human mesenchymal stem cells. Cancer Res. 2008;68:4331–9. 18519693 10.1158/0008-5472.CAN-08-0943 PMC2725025

[7] Lee KW, Yeo SY, Sung CO, Kim SH. Twist1 Is a Key Regulator of Cancer-Associated Fibroblasts. Cancer Res. 2015;75:73–85. 25368021 10.1158/0008-5472.CAN-14-0350

[8] Karagiannis GS, Poutahidis T, Erdman SE, Kirsch R, Riddell RH, Diamandis EP. Cancer-Associated Fibroblasts Drive the Progression of Metastasis through both Paracrine and Mechanical Pressure on Cancer Tissue. Mol Cancer Res. 2012;10:1403–18. 23024188 10.1158/1541-7786.MCR-12-0307 PMC4399759

[9] Sahai E, Astsaturov I, Cukierman E, DeNardo DG, Egeblad M, Evans RM, et al. A framework for advancing our understanding of cancer-associated fibroblasts. Nat Rev Cancer. 2020;20:174–86. 31980749 10.1038/s41568-019-0238-1 PMC7046529

[10] Fang Z, Meng Q, Xu J, Wang W, Zhang B, Liu J, et al. Signaling pathways in cancer-associated fibroblasts: recent advances and future perspectives. Cancer Commun (Lond). 2023;43:3–41. 36424360 10.1002/cac2.12392 PMC9859735

[11] Chen Y, Zhu S, Liu T, Zhang S, Lu J, Fan W, et al. Epithelial cells activate fibroblasts to promote esophageal cancer development. Cancer Cell. 2023;41:903–18 e8. 36963399 10.1016/j.ccell.2023.03.001

[12] Saxena N, Chakraborty S, Dutta S, Bhardwaj G, Karnik N, Shetty O, et al. Stiffness-dependent MSC homing and differentiation into CAFs - implications for breast cancer invasion. J Cell Sci. 2024;137:jcs261145. 38108421 10.1242/jcs.261145

[13] Itoh G, Takagane K, Fukushi Y, Kuriyama S, Umakoshi M, Goto A, et al. Cancer-associated fibroblasts educate normal fibroblasts to facilitate cancer cell spreading and T-cell suppression. Mol Oncol. 2022;16:166–87. 34379869 10.1002/1878-0261.13077 PMC8732346

[14] Sanchez-Martin L, Estecha A, Samaniego R, Sanchez-Ramon S, Vega MA, Sanchez-Mateos P. The chemokine CXCL12 regulates monocyte-macrophage differentiation and RUNX3 expression. Blood. 2011;117:88–97. 20930067 10.1182/blood-2009-12-258186

[15] Balabanian K, Lagane B, Infantino S, Chow KYC, Harriague J, Moepps B, et al. The chemokine SDF-1/CXCL12 binds to and signals through the orphan receptor RDC1 in T lymphocytes. J Biol Chem. 2005;280:35760–6. 16107333 10.1074/jbc.M508234200

[16] Saha A, Ahn S, Blando J, Su F, Kolonin MG, DiGiovanni J. Proinflammatory CXCL12-CXCR4/CXCR7 Signaling Axis Drives Myc-Induced Prostate Cancer in Obese Mice. Cancer Res. 2017;77:5158–68. 28687617 10.1158/0008-5472.CAN-17-0284 PMC5600849

[17] Jiang H, Ge H, Shi Y, Yuan F, Yue H. CAFs secrete CXCL12 to accelerate the progression and cisplatin resistance of colorectal cancer through promoting M2 polarization of macrophages. Med Oncol. 2023;40:90. 36737590 10.1007/s12032-023-01953-7

[18] Liu Q, Yu B, Tian Y, Dan J, Luo Y, Wu X. P53 Mutant p53(N236S) Regulates Cancer-Associated Fibroblasts Properties Through Stat3 Pathway. Onco Targets Ther. 2020;13:1355–63. 32104002 10.2147/OTT.S229065 PMC7027832

[19] Liotta LA, Kohn EC. The microenvironment of the tumour-host interface. Nature. 2001;411:375–9. 11357145 10.1038/35077241

[20] Zheng H, Liu H, Ge Y, Wang X. Integrated single-cell and bulk RNA sequencing analysis identifies a cancer associated fibroblast-related signature for predicting prognosis and therapeutic responses in colorectal cancer. Cancer Cell Int. 2021;21:552. 34670584 10.1186/s12935-021-02252-9 PMC8529760

[21] Yasumoto K, Koizumi K, Kawashima A, Saitoh Y, Arita Y, Shinohara K, et al. Role of the CXCL12/CXCR4 axis in peritoneal carcinomatosis of gastric cancer. Cancer Res. 2006;66:2181–7. 16489019 10.1158/0008-5472.CAN-05-3393

[22] Qin Y, Wang F, Ni H, Liu Y, Yin Y, Zhou X, et al. Cancer-associated fibroblasts in gastric cancer affect malignant progression via the CXCL12-CXCR4 axis. J Cancer. 2021;12:3011–23. 33854601 10.7150/jca.49707 PMC8040897

[23] Sugihara H, Ishimoto T, Yasuda T, Izumi D, Eto K, Sawayama H, et al. Cancer-associated fibroblast-derived CXCL12 causes tumor progression in adenocarcinoma of the esophagogastric junction. Med Oncol. 2015;32:618. 25920609 10.1007/s12032-015-0618-7

[24] Liu LD, Dong CH, Shi HJ, Zhao HL, Wang LC, Ma SH, et al. A novel type II membrane receptor up-regulated by IFN-alpha in fibroblasts functions in cell proliferation through the JAK-STAT signalling pathway. Cell Prolif. 2006;39:93–103. 16542345 10.1111/j.1365-2184.2006.00373.x PMC6496284

[25] Santagata S, Ierano C, Trotta AM, Capiluongo A, Auletta F, Guardascione G, et al. CXCR4 and CXCR7 Signaling Pathways: A Focus on the Cross-Talk Between Cancer Cells and Tumor Microenvironment. Front Oncol. 2021;11:591386. 33937018 10.3389/fonc.2021.591386 PMC8082172

[26] Hendrayani SF, Al-Khalaf HH, Aboussekhra A. The Cytokine IL-6 Reactivates Breast Stromal Fibroblasts through Transcription Factor STAT3-dependent Up-regulation of the RNA-binding Protein AUF1. J Biol Chem. 2014;289:30962–76. 25231991 10.1074/jbc.M114.594044 PMC4223303

[27] Biffi G, Oni TE, Spielman B, Hao Y, Elyada E, Park Y, et al. IL1-Induced JAK/STAT Signaling Is Antagonized by TGFbeta to Shape CAF Heterogeneity in Pancreatic Ductal Adenocarcinoma. Cancer Discov. 2019;9:282–301. 30366930 10.1158/2159-8290.CD-18-0710 PMC6368881

[28] Tang Y, Lu Y, Chen Y, Luo L, Cai L, Peng B, et al. Pre-metastatic niche triggers SDF-1/CXCR4 axis and promotes organ colonisation by hepatocellular circulating tumour cells via downregulation of Prrx1. J Exp Clin Cancer Res. 2019;38:473. 31752959 10.1186/s13046-019-1475-6 PMC6873584

[29] Ramteke A, Ting H, Agarwal C, Mateen S, Somasagara R, Hussain A, et al. Exosomes secreted under hypoxia enhance invasiveness and stemness of prostate cancer cells by targeting adherens junction molecules. Mol Carcinog. 2015;54:554–65. 24347249 10.1002/mc.22124 PMC4706761

[30] Martinez-Ordonez A, Seoane S, Cabezas P, Eiro N, Sendon-Lago J, Macia M, et al. Breast cancer metastasis to liver and lung is facilitated by Pit-1-CXCL12-CXCR4 axis. Oncogene. 2018;37:1430–44. 29321662 10.1038/s41388-017-0036-8

[31] Sansone P, Bromberg J. Targeting the interleukin-6/Jak/stat pathway in human malignancies. J Clin Oncol. 2012;30:1005–14. 22355058 10.1200/JCO.2010.31.8907 PMC3341105

[32] Avalle L, Raggi L, Monteleone E, Savino A, Viavattene D, Statello L, et al. STAT3 induces breast cancer growth via ANGPTL4, MMP13 and STC1 secretion by cancer associated fibroblasts. Oncogene. 2022;41:1456–67. 35042959 10.1038/s41388-021-02172-y

[33] Zhang C, Fei Y, Wang H, Hu S, Liu C, Hu R, et al. CAFs orchestrates tumor immune microenvironment-A new target in cancer therapy? Front Pharm. 2023;14:1113378. 10.3389/fphar.2023.1113378 PMC10064291 37007004

[34] Zindy F, Quelle DE, Roussel MF, Sherr CJ. Expression of the p16INK4a tumor suppressor versus other INK4 family members during mouse development and aging. Oncogene. 1997;15:203–11. 9244355 10.1038/sj.onc.1201178

[35] Tang Z, Kang B, Li C, Chen T, Zhang Z. GEPIA2: an enhanced web server for large-scale expression profiling and interactive analysis. Nucleic Acids Res. 2019;47:W556–W560. 31114875 10.1093/nar/gkz430 PMC6602440

