# Supplementary material for: CXCL12 alone is enough to Reprogram Normal Fibroblasts into Cancer-Associated Fibroblasts
Source: Cell Death Discov. 2025 Apr 8;11:156. doi: 10.1038/s41420-025-02420-0 (PMC11978793; doi:10.1038/s41420-025-02420-0)

**Figure S1. p53 mutations in CAFs.**

**
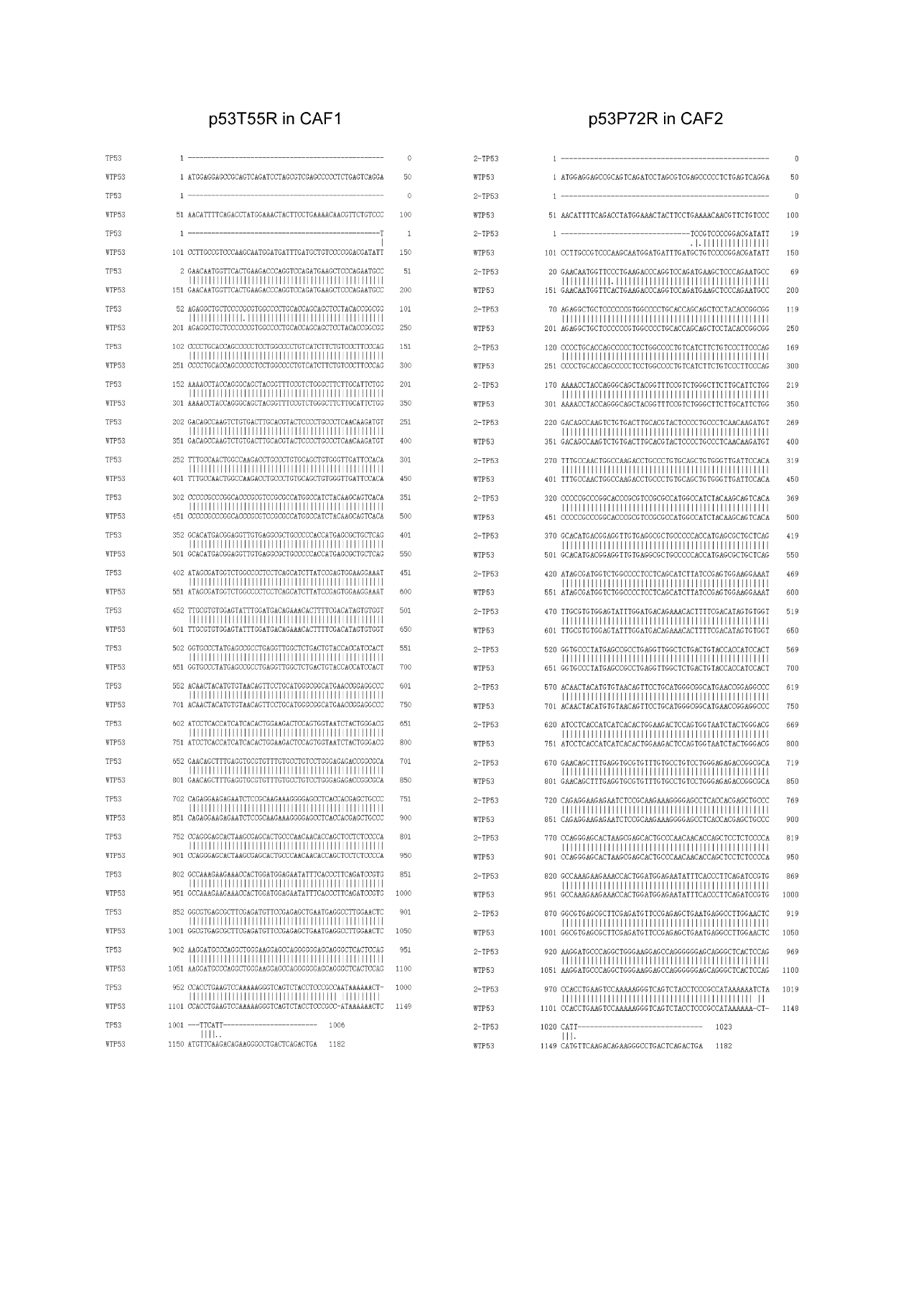
**

**Figure S2. p53S Fibroblast display CAFs properties**

1. Western blot analysis for α-SMA and Vimentin expression in NFs(*p53^+/+^*), *p53^-/-^* fibroblast and p53S-CAFs. Diagrams represent α-SMA and Vimentin protein levels as means ± SEM of densitometric quantifications of three independent experiments (n = 3) normalized to Vinculin. Statistical analysis. *, p<0.05.
2. Microscopic examination of NFs, *p53^-/-^* fibroblast and p53S-CAFs; Immunofluorescence analysis of CAFs marker α-SMA and Vimentin expression and the percentage of incorporated EdU positive population in normal fibroblast, *p53^-/-^* fibroblast and p53S-CAFs. **, p<0.005, ***, p<0.0005.
3. Transwell migration assay of normal fibroblast, *p53^-/-^* fibroblast and p53S-CAFs. Average migration ±SEM from two independent experiments performed(n=3). *, p<0.05. **, p<0.005.
4. The migratory and invasive capacities of LLC cells cocultured with CM from NFs, *p53^-/-^* fibroblasts, and p53S-CAFs were determined by transwell assay, and statistical analysis was performed. **p < 0.01, ***p < 0.001. Scale bar = 200 μm


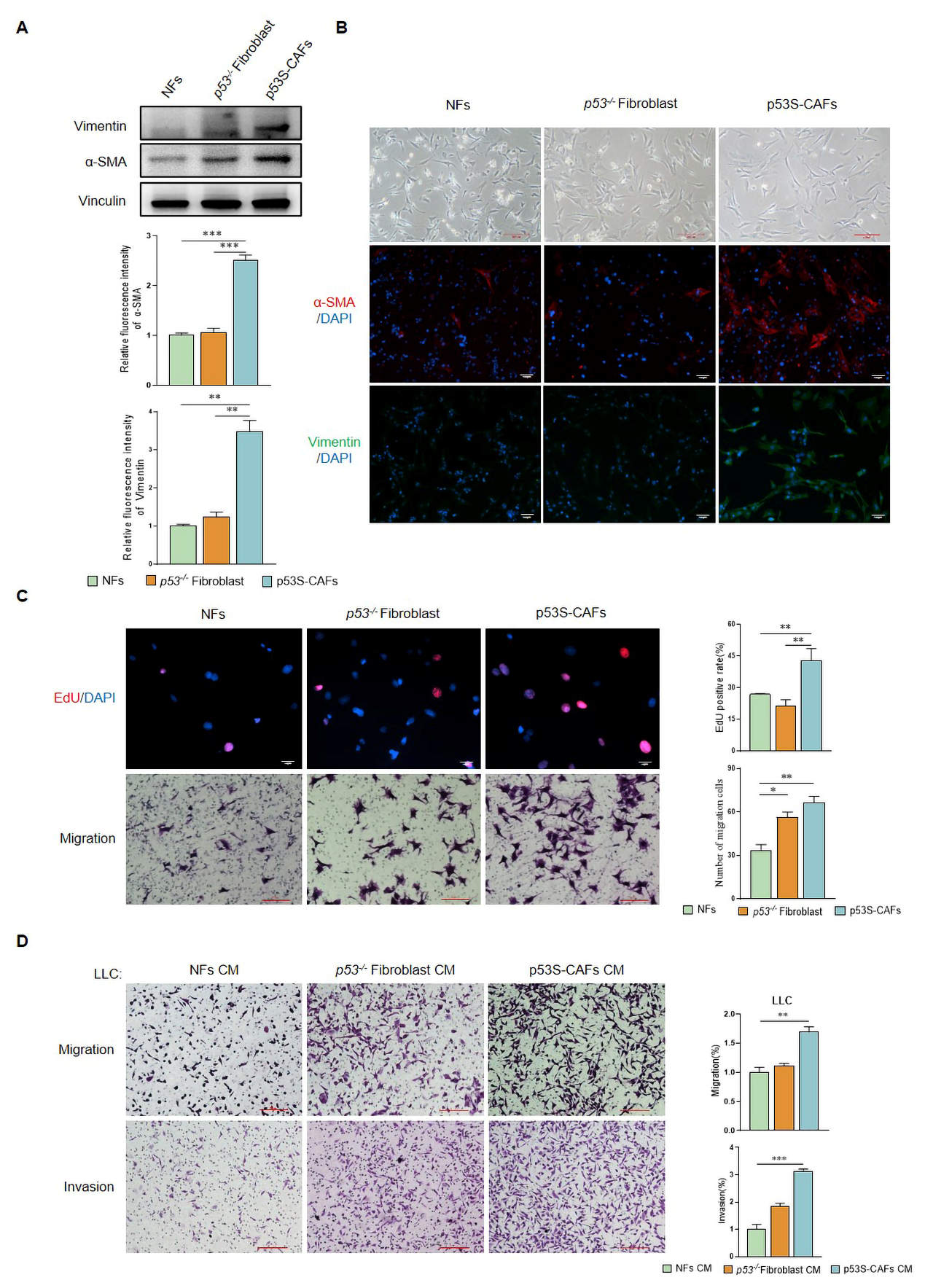


**Figure S3.** Transwell co-culture assay was employed to evaluate the pro-migratory capacity of normal fibroblasts, CEFs-12 (normal fibroblasts treated with CXCL12), CEFs (normal fibroblasts treated with p53S-CAFs conditioned medium), and CEFs+AMD3100 towards cancer cells Hela and U2OS. The results include representative images of cell migration and statistical data. **, p<0.005, ***, p<0.0005. Scale bar = 200 μm


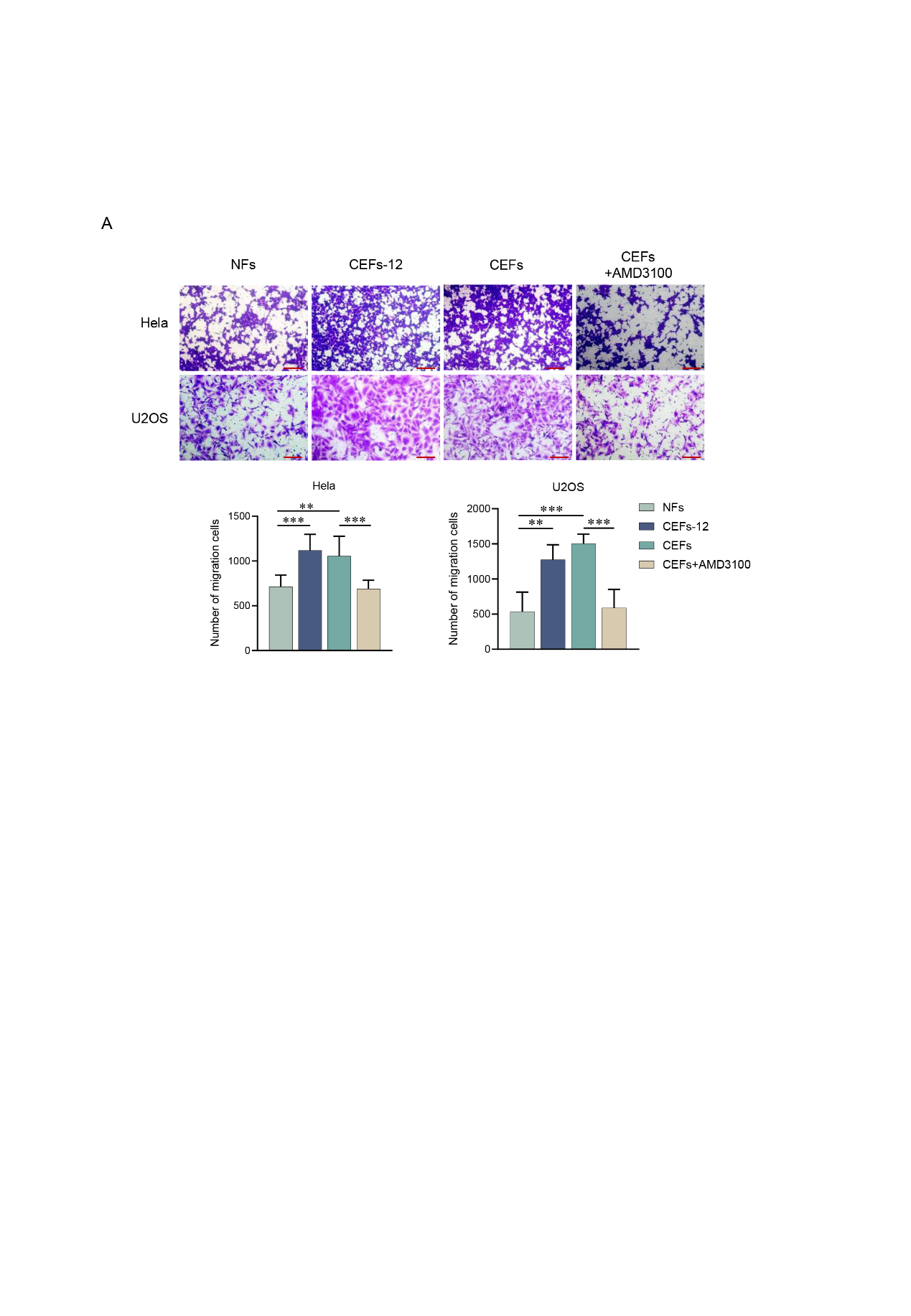

Supplement: Supplementary file 1 — Supplementary Figure [file 41420_2025_2420_MOESM1_ESM.docx]
