# Supplementary material for: CXCL12 alone is enough to Reprogram Normal Fibroblasts into Cancer-Associated Fibroblasts
Source: Cell Death Discov. 2025 Apr 8;11:156. doi: 10.1038/s41420-025-02420-0 (PMC11978793; doi:10.1038/s41420-025-02420-0)

Figure 1-C

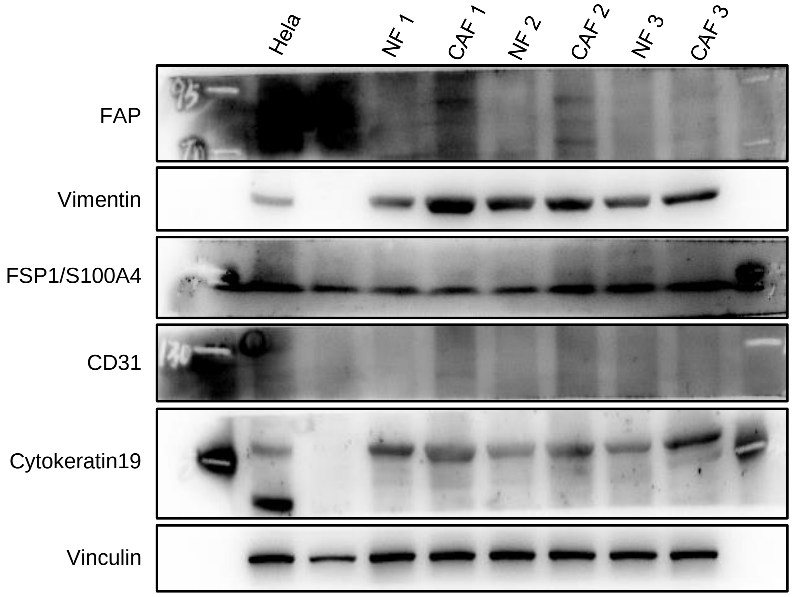

Figure 1-E

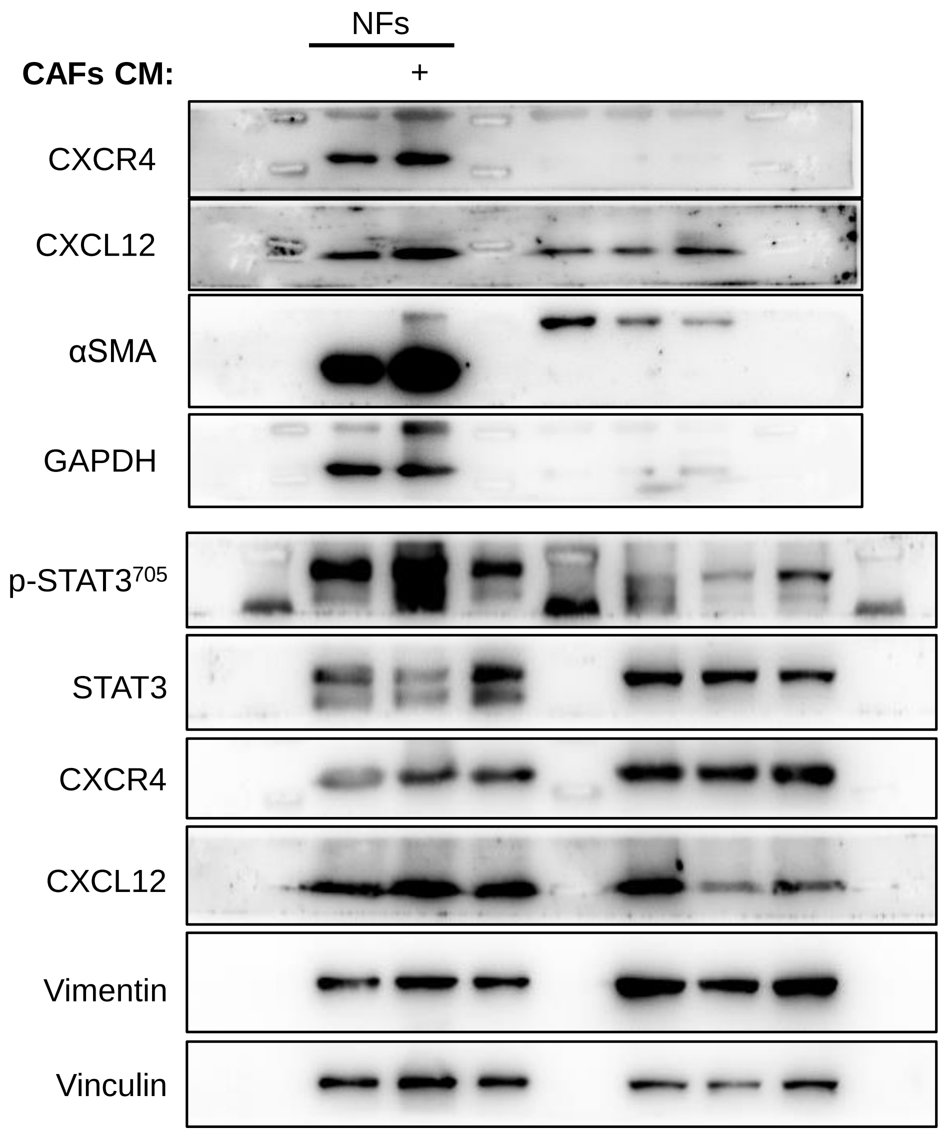

Figure 2-A

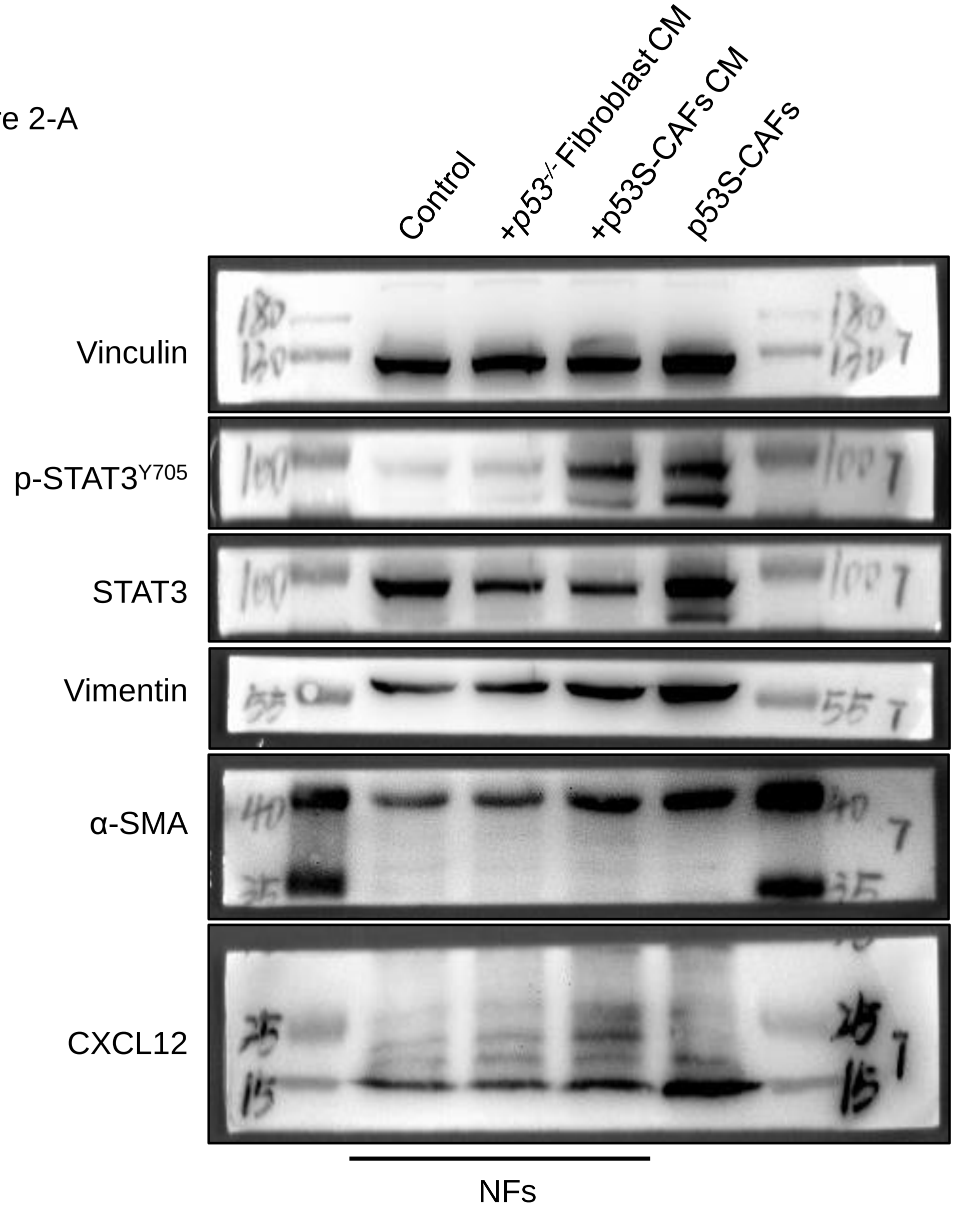

Figure 4-A

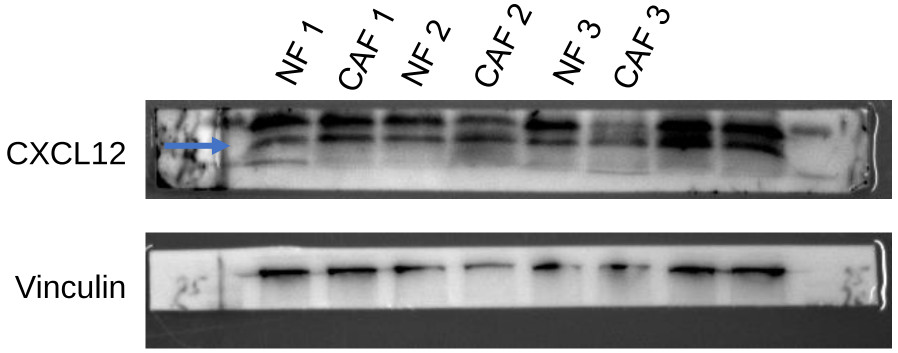

Figure 5-A

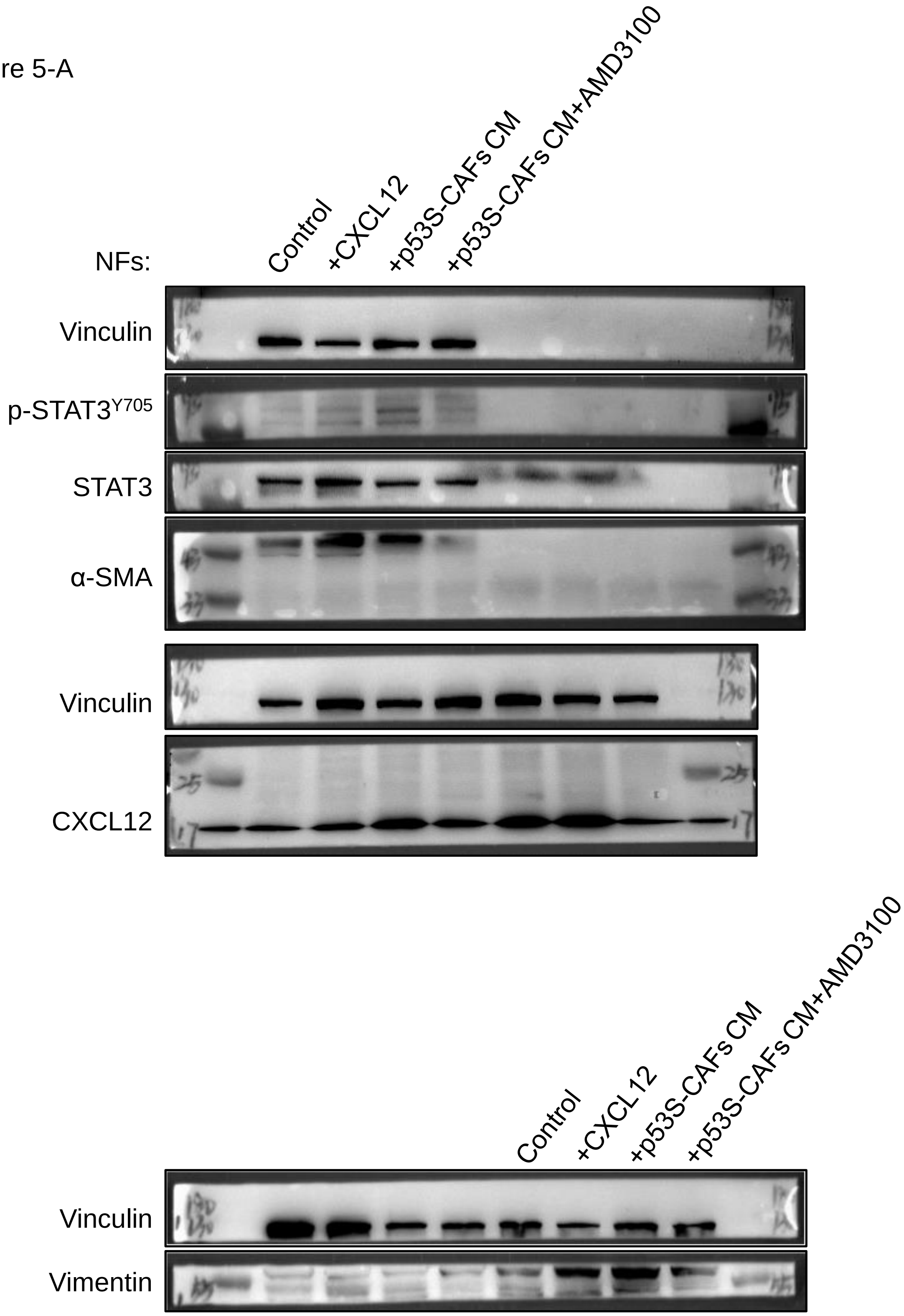
NFs:
Control
+CXCL12
+p53S-CAFs CM
+p53S-CAFs CM+AMD3100
Vinculin
p-STAT3Y705
STAT3
α-SMA
Vinculin
CXCL12
Control
+CXCL12
+p53S-CAFs CM
+p53S-CAFs CM+AMD3100
Vinculin
Vimentin

Figure 6-A

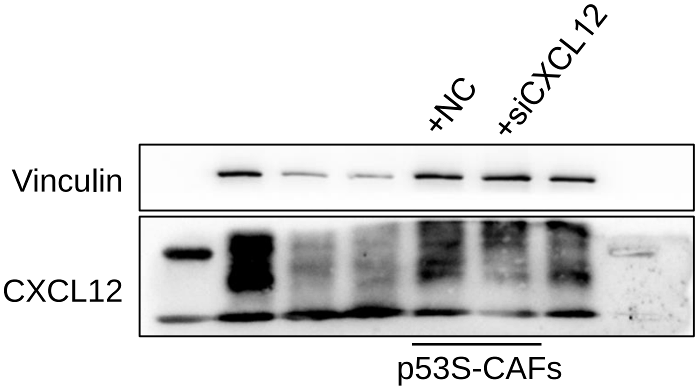

Figure 6-B

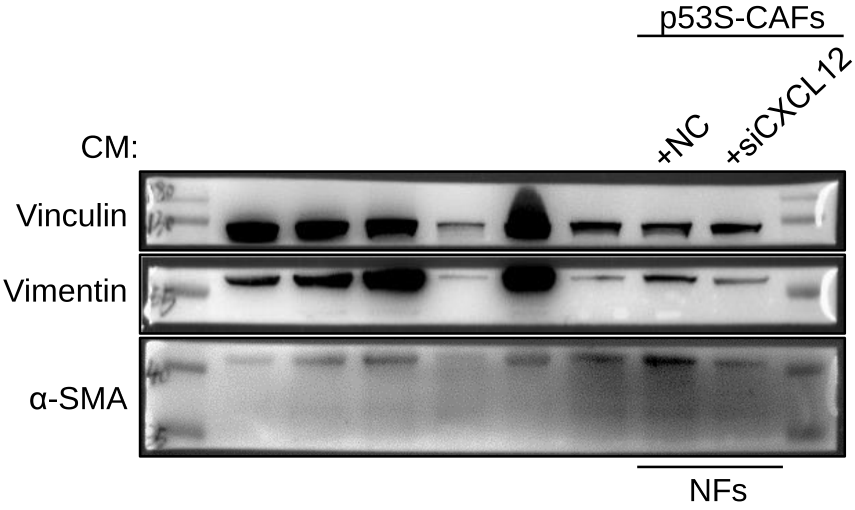

Figure 7-A

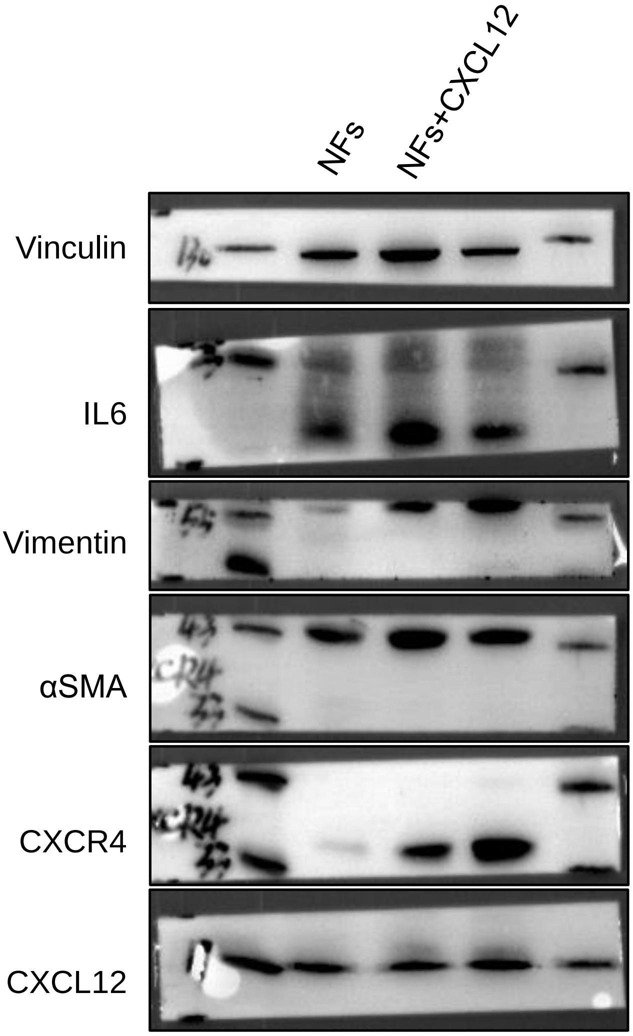

Figure 7-A

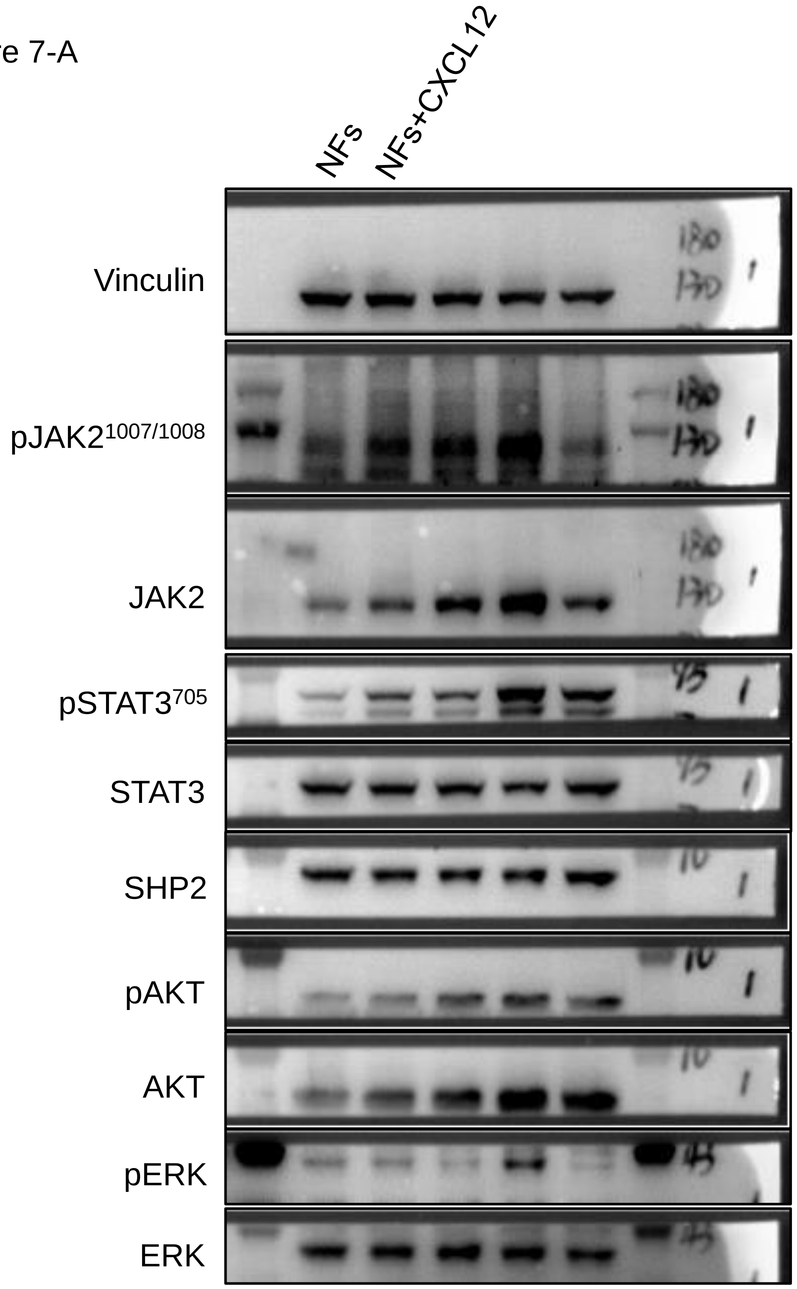

Figure 7-B

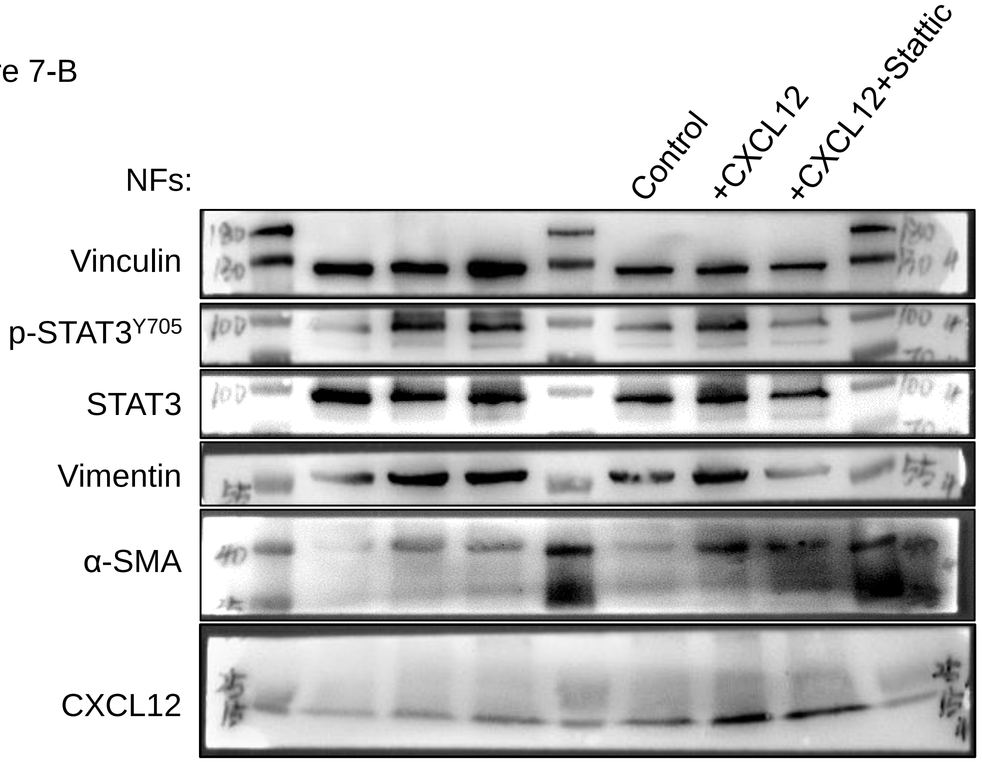

Figure 7-E

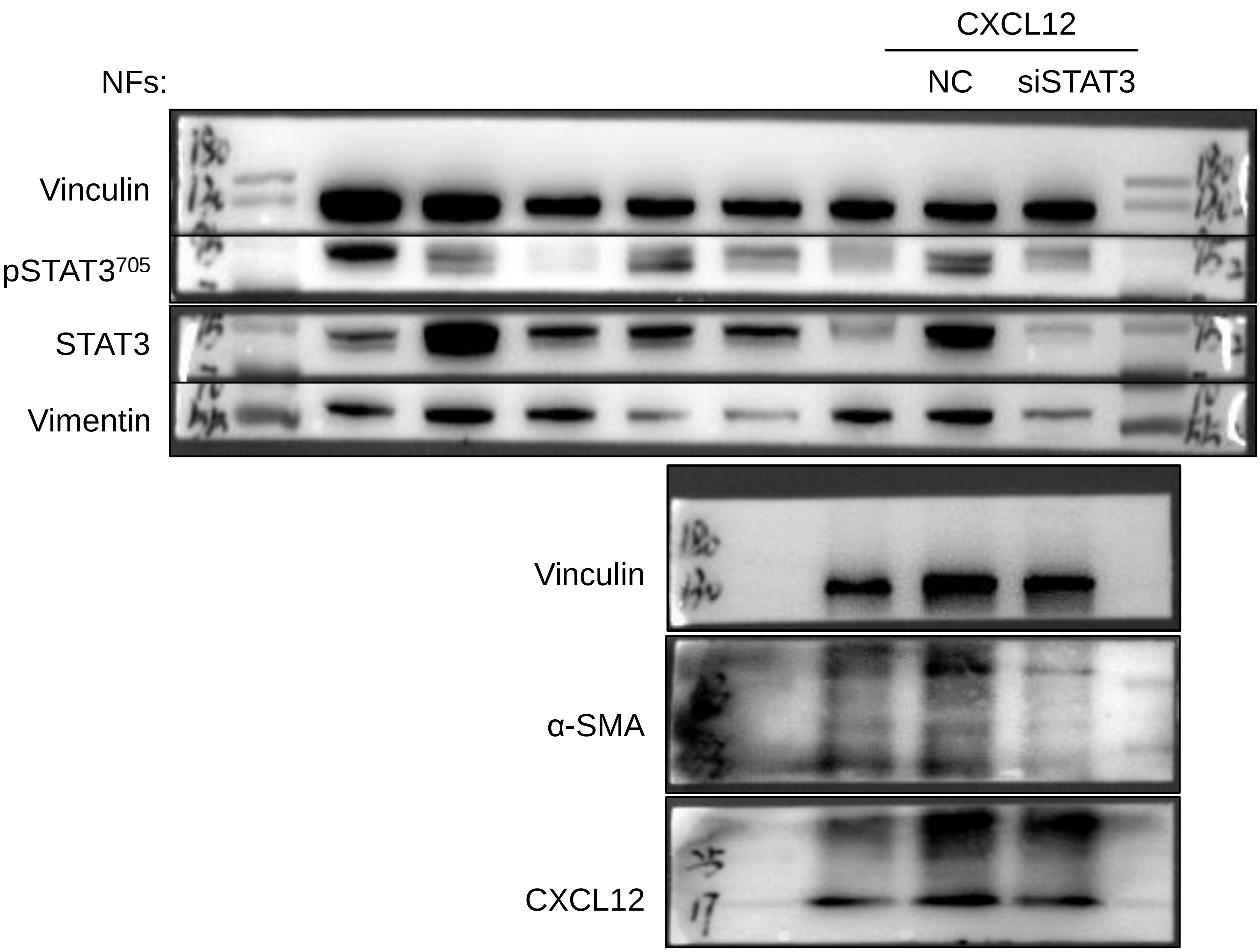

Figure S1-A

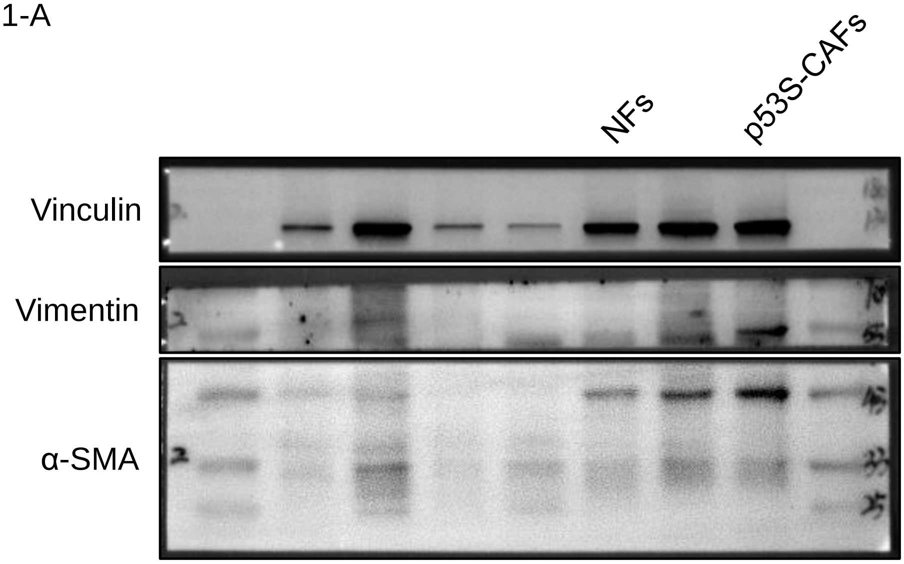

Supplement: Supplementary file 2 — Original Images for BlotsGels [file 41420_2025_2420_MOESM2_ESM.pdf]
